# The miRNAome of *Catharanthus roseus*: identification, expression analysis, and potential roles of microRNAs in regulation of terpenoid indole alkaloid biosynthesis

**DOI:** 10.1038/srep43027

**Published:** 2017-02-22

**Authors:** Ethan M. Shen, Sanjay K. Singh, Jayadri S. Ghosh, Barunava Patra, Priyanka Paul, Ling Yuan, Sitakanta Pattanaik

**Affiliations:** 1 Department of Plant and Soil Sciences, University of Kentucky, 1401 University Drive, Lexington, KY 40546, USA; 2 Math, Science, and Technology Center, Paul Laurence Dunbar High School, 1600 Man o’ War Boulevard, Lexington, KY 40513, USA.

## Abstract

MicroRNAs (miRNAs) regulate numerous crucial biological processes in plants. However, information is limited on their involvement in the biosynthesis of specialized metabolites in plants, including *Catharanthus roseus* that produces a number of pharmaceutically valuable, bioactive terpenoid indole alkaloids (TIAs). Using small RNA-sequencing, we identified 181 conserved and 173 novel miRNAs (cro-miRNAs) in *C. roseus* seedlings. Genome-wide expression analysis revealed that a set of cro-miRNAs are differentially regulated in response to methyl jasmonate (MeJA). *In silico* target prediction identified 519 potential cro-miRNA targets that include several auxin response factors (ARFs). The presence of cleaved transcripts of miRNA-targeted *ARFs* in *C. roseus* cells was confirmed by Poly(A) Polymerase-Mediated Rapid Amplification of cDNA Ends (PPM-RACE). We showed that auxin (indole acetic acid, IAA) repressed the expression of key TIA pathway genes in *C. roseus* seedlings. Moreover, we demonstrated that a miRNA-regulated ARF, CrARF16, binds to the promoters of key TIA pathway genes and repress their expression. The *C. roseus* miRNAome reported here provides a comprehensive account of the cro-miRNA populations, as well as their abundance and expression profiles in response to MeJA. In addition, our findings underscore the importance of miRNAs in posttranscriptional control of the biosynthesis of specialized metabolites.

As sessile organisms, plants have evolved unique mechanisms to defend themselves in adverse environmental conditions. Plants synthesize thousands of specialized metabolites that play unique roles in plant growth, development, and defense. Many of these metabolites are beneficial for humans. *Catharanthus roseus* (L.) G. Don, commonly known as Madagascar periwinkle, synthesizes over 130 terpenoid indole alkaloids (TIA), including the pharmaceutically important anti-neoplastic compounds, vinblastine and vincristine^1^. Biosynthesis of TIAs is highly complex, involving multiple sub-cellular compartments. Tryptamine, derived from the indole branch, and secologanin, from the seco-iridoid branch, are condensed to form the first TIA, strictosidine, catalyzed by STRICTOSIDINE SYNTHASE (STR). The biosynthesis of TIAs is induced by a number of factors, including fungal elicitors^2^, UV light^3^, wounding^4^, cold^5^, and drought stress^6^. The phytohormone jasmonic acid (JA) and its methyl ester, methyl jasmonate (MeJA), are major elicitors of TIA biosynthesis in *C. roseus*. JA-responsive expression of TIA pathway genes are positively regulated by transcription factors (TFs), such as the JA-responsive AP2/ERFs, ORCA2^7^, ORCA3^8^, ORCA4 and ORCA5^9^, as well as the bHLH factors, CrMYC2^10^, BIS1^11^, and BIS2^12^, and the WRKY TF, CrWRKY1^13^. Several repressors, including the G-box binding basic leucine-zipper (bZIP) factors, GBF1 and GBF2^14^, and zinc finger proteins, ZCT1, ZCT2, and ZCT3^15^,^16^, negatively regulate the expression of TIA pathway genes in *C. roseus*. Other phytohormones, such as cytokinin (CK) and auxin, also affect TIA biosynthesis. In *C. roseus* cell suspension culture, CK enhances the accumulation of alkaloids^17^, whereas auxin negatively regulates the expression of key TIA biosynthesis genes, including *TRYPTOPHAN DECARBOXYLASE (TDC*) and *STR*^18^,^19^. However, the molecular mechanism of auxin-mediated regulation of the TIA pathway is unknown. Auxin is an essential phytohormone that plays pivotal roles in plant growth and development. Auxin regulates gene expression through a signal transduction pathway which includes the F-box protein TRANSPORT INHIBITOR RESPONSE 1/AUXIN SIGNALING F-BOX PROTEINS (TIR1/AFB), AUXIN-RESPONSE FACTOR (ARF), and Auxin/INDOLE-3-ACETIC ACID (Aux/IAA) proteins. ARFs are DNA binding proteins that recognize auxin-responsive (AuxRE) elements in target promoters to activate or repress expression^20^. In Arabidopsis, expression of *ARFs* are regulated posttranscriptionally by microRNAs (miRNAs)^21^,^22^.

miRNAs comprise a major class of endogenous non-coding small regulatory RNAs approximately 21 to 24 nucleotides in length. They are present in a variety of organisms from algae to plants^23^. In plants, mature miRNAs are processed from primary transcripts by DICER-LIKE 1 RNase (DCL1), and subsequently loaded onto the ARGONAUTE (AGO) protein(s) to form RNA-induced silencing complexes (RISCs)^24^. The miRNA-loaded RISC binds to the target mRNA in a sequence-specific manner to either degrade the mRNA^25^ or prevent it from translation^26^. Many plant miRNAs are evolutionarily conserved among plant species^27^,^28^. miRNAs are involved in numerous biological processes, including plant hormone homeostasis^29^, root development^30^,^31^, leaf morphogenesis^32^,^33^, flower development^34^, and embryogenesis^35^,^36^. However, information on the involvement of miRNA in regulation of specialized metabolite biosynthesis is limited^37^,^38^,^39^. Whether miRNAs are involved in regulating TIA biosynthesis in *C. roseus* has not been extensively investigated.

A previous study has identified 81 conserved and 7 novel miRNAs in *C. roseus* seedlings using deep sequencing^40^. The number of identified miRNAs in the report is considerably lower than the average number of miRNAs in plants studied thus far. Moreover, as the *C. roseus* genome sequence was unavailable during the previous study, the tomato genome was used as the reference to predict novel miRNAs. Here, we systematically identified miRNAs in *C. roseus* by sequencing four small RNA libraries from the control and JA-treated seedlings. We identified a total of 354 *C. roseus* miRNAs, including 181 conserved and 173 novel miRNAs. All identified miRNAs were mapped to the recently available *C. roseus* reference genome^41^. Expression of selected miRNAs and their potential targets were validated using quantitative reverse transcription PCR (qRT-PCR). In addition, we predicted a set of MeJA-responsive miRNAs that target a group of ARFs in *C. roseus*. Cleaved ARF transcripts in *C. roseus* cells were identified by Poly(A) Polymerase-Mediated Rapid Amplification of cDNA Ends (PPM-RACE). We hypothesized that ARFs bind to TIA pathway gene promoters to repress their activity, and at least some of these ARFs are regulated by miRNAs. We demonstrated that a JA-responsive, miRNA regulated ARF, CrARF16, binds to key TIA pathway gene promoters to repress their expression. Our findings provide a comprehensive account of the *C. roseus* miRNAome, and suggest that miRNAs and ARFs are involved in the regulation of TIA biosynthesis.

## Results

### Small RNA populations in *C. roseus* seedlings

**Figure 1:**
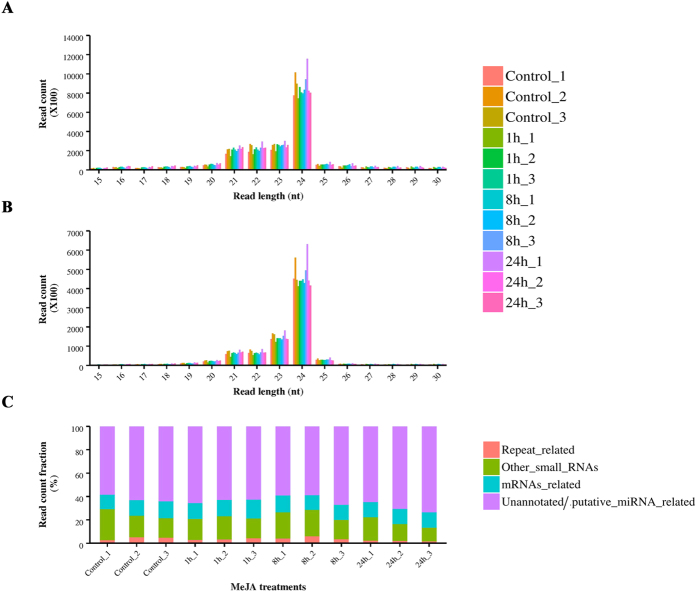
Length distribution and different classes of sequence reads identified in the small RNA libraries of *C. roseus*. **(A)** Size distribution of the total reads. **(B)** Size distribution of unique reads. **(C)** Distribution of different classes of sequence reads derived from the small RNA libraries. Putative miRNAs and un-annotated sRNAs constitute 60–75% of the total cleaned reads in all twelve libraries. Each small RNA library (control, 1 h-, 8 h-, or 24 h-MeJA treated) is represented by three biological replicates. *C. roseus* seedlings treated with 100 μM MeJA for 1 h, 8 h, or 24 h were used for RNA isolation and library preparation. Mock-treated seedlings served as control.

A total of 12 small RNA (sRNA) libraries were constructed from the control and MeJA-treated *C. roseus* seedlings (Supplementary Fig. S1). Sequencing of small RNA libraries, from control and seedlings treated with MeJA for 1 h, 8 h, and 24 h, produced approximately 76.5 million (M), 76.8 M, 79 M, and 84.5 M raw reads, respectively (Supplementary Table S1). Among the raw reads, 88–91% were found to contain adapter sequences and thus chosen for further analysis. After removing low-quality reads and trimming the adaptor sequences, approximately 64.1 M, 65.5 M, 69.8 M, and 73 M clean reads were obtained for the control, 1 h-, 8 h-, and 24 h-MeJA-treated libraries, respectively (Supplementary Table S1). A total of 60.7 M (control), 62.2 M (1 h), 66.6 M (8 h), and 69.3 M (24 h) sequences were successfully mapped to the *C. roseus* reference genome. Sequences mapped to the *C. roseus* reference genome were further subjected to removal of transfer and ribosomal RNAs (t/rRNAs). Size distribution analysis of the sRNA sequences after filtering showed that all libraries exhibit similar distribution in length, with the most abundant class being 24 nt (Fig. 1A). After removal of redundant reads, the 24 nt reads remained most abundant, followed by the 23 nt reads (Fig. 1B). Overall, putative miRNAs and un-annotated sRNAs constitute 60–75% of the total cleaned reads in all twelve libraries (Fig. 1C).

### Conserved miRNAs in *C. roseus*

**Figure 2:**
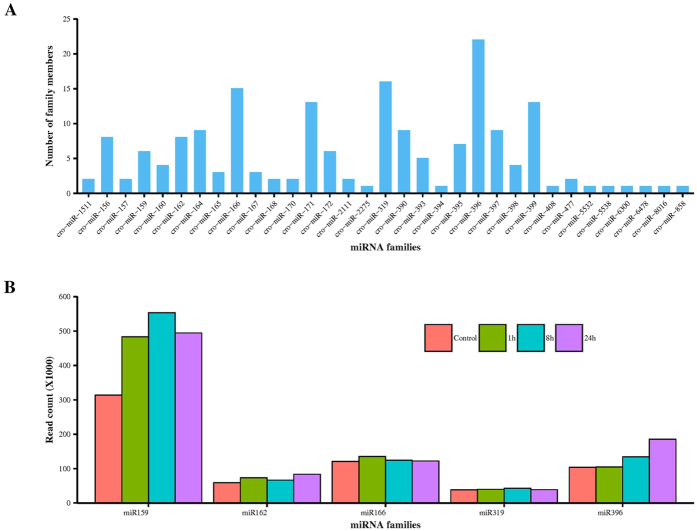
Number of members and abundance of known miRNA families identified in *C. roseus.* (**A**) Number of members in each conserved miRNA family in *C. roseus*. (**B**) Sequence read counts of top five conserved miRNA families in the small RNA libraries. The transcript abundance of each family in the control, as well as 1 h-, 8 h-, and 24 h-MeJA treated library is shown.

To identify conserved miRNAs in *C. roseus*, the unique sequences were aligned to the mature sequences of known miRNAs deposited in the miRBase database^42^. A total of 181 conserved miRNAs, representing 33 miRNA families, were identified in *C. roseus* (Supplementary Table S2). Conserved miRNA varied from 18 nt to 24 nt in length. Most of the conserved miRNA families reported by Prakash *et al*.^40^ are also present in our dataset. However, we could not detect miR169, miR530, miR828, miR2199, miR5139, miR5368, and miR6173 in our dataset; on the other hand, we detected miR2275, miR5532, miR5538, miR6300, miR6478, miR8016, miR858, miR397, miR390, miR393, miR394, and miR395, that were not reported by Prakash *et al*.^40^. In addition, 65 precursor sequences, representing 28 conserved miRNAs, were also predicted from the *C. roseus* genome (Supplementary Table S3). Several cro-miRNA families were found to be encoded by multiple loci in the *C. roseus* genome. For instance, cro-miR159 is encoded by two different loci (cro_scaffold_3041204 and cro_scaffold_3039079). Of the 65 identified precursor sequences, cro-miR159, -miR396, and -miR166 showed high abundance, having an average normalized count (NC) >1,000 (Supplementary Table S4), similar to those observed in other plant species, including radish and banana^43^,^44^. Cro-miR2275, -miR477, and -miR2111 were found to be least abundant, with an average NC less than 10. A considerable variation in expression was also noticed among loci coding for the same miRNA family (Supplementary Table S4). For instance, the average NC for the cro-miR319 family varied from 15–542. Similar variations in read abundance were also observed among members of the miRNA families such as cro-miR396 (1–1440), -miR395 (1–94), -miR171 (0.3–44), and -miR156 (1–194) (Supplementary Table S4). The average minimal folding free energy (MFE) value of the miRNA precursors was −49.2 kcal mol^−1^; and the length of the precursors ranged from 75 nt to 377 nt, with an average length of 166 nt (Supplementary Table S3). The predicted secondary structures of the known miRNA precursors and locations of mature miRNAs in precursors were shown in Supplementary Fig. S2. A significant difference in the number of members was recorded for each conserved miRNA family (Fig. 2A). The largest family identified was cro-miR396 with 22 members, followed by cro-miR319 and -miR166, with 16 and 15 members, respectively. Of the remaining 30 families, 21 families were represented by 2–13 members while 9 were represented by a single member (Fig. 2A). Considerable variation in expression was observed among the individual families (Fig. 2B), and also among members of the same miRNA family. For instance, of the 6 members of the cro-miR159 family, cro-miR159a showed highest abundance (average NC > 1,000) while cro-miR159c was found to be least abundant (average NC < 1.0) (Supplementary Table S5).

### Novel *C. roseus* miRNAs

A total of 173 novel *C. roseus* miRNAs were predicted based on established prediction criteria^45^,^46^ (Supplementary Tables S6 and S7). The length of these novel miRNA precursors, ranging from 55–491 nt and averaging in 137 nt, agreed with the commonly observed length of miRNA precursors in plants^47^. The average minimal folding free energy (MFE) value of these miRNA precursors was −43.2 kcal mol^−1^. The predicted secondary structures of these novel miRNA precursors and locations of mature miRNAs in precursors are shown in Supplementary Fig. S3. The sequence read abundance of the majority of *C. roseus* novel miRNAs were low compared to the conserved cro-miRNAs (Supplementary Table S8). A considerable variation in expression was also observed in *C. roseus*-specific miRNAs. Of the 173 *C. roseus*-specific miRNAs, the NC for 22 miRNAs were less than 10, whereas that for 134 miRNAs ranged from 10 to 200. Seventeen abundant novel *C. roseus*-specific miRNAs, including cro-novel-35, cro-novel-51, and cro-novel-56, have NC of more than 200 (Supplementary Table S8). The authenticity of predicted novel miRNAs is usually supported by the existence of complementary sequences^46^. In *C. roseus*, 162 of the 173 novel miRNAs have complementary miRNAs.

### MeJA-responsive miRNAs in *C. roseus* seedlings

**Figure 3:**
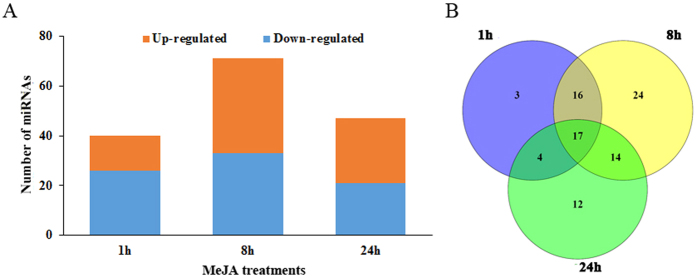
Differential expression analysis of miRNAs in *C. roseus* seedlings. **(A)** Numbers of up- and downregulated miRNAs in *C. roseus* seedlings after MeJA-treatment for 1 h, 8 h, or 24 h (**B**) Venn diagram showing the overlap of differentially expressed miRNAs (DEMs) between the three time points.

We analyzed the small RNA libraries to identify differentially expressed miRNAs (DEMs) in *C. roseus* seedlings with or without MeJA treatment. We identified 40 (14 upregulated and 26 downregulated), 71 (38 upregulated and 33 downregulated), and 47 (26 upregulated and 21 downregulated) JA-responsive miRNAs after 1 h, 8 h, and 24 h of MeJA treatment, respectively (Fig. 3A and Supplementary Table S9). Thirty-six conserved miRNAs, belonging to 14 miRNA families, such as cro-miR160 and -miR164, were differentially expressed in at least one time-point. Additionally, expression of 11 miRNAs, cro-miR319a-5p, cro-miR164b, cro-miR390b-3p-1, cro-miR164a, cro-miR393d, cro-novel-62, cro-novel-92, cro-novel-73, cro-novel-65, cro-novel-10, and cro-novel-63, were increased after MeJA treatment in all time-points. In contrast, 6 miRNAs, cro-novel-64, cro-novel-54, cro-novel-130, cro-miR398f, cro-miR319a-3p.2–3p, and cro-miR319a, were downregulated at all time-points.

**Figure 4:**
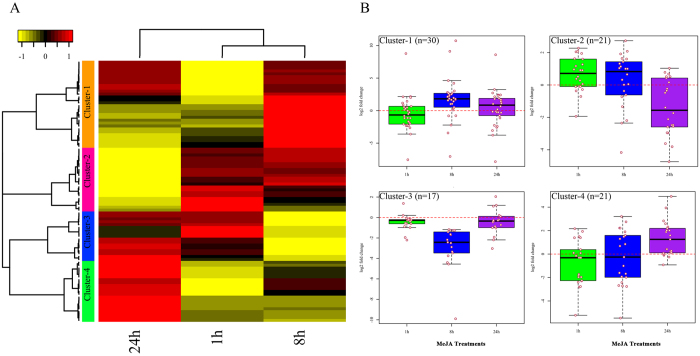
Hierarchical cluster analysis of DEMs in *C. roseus* seedlings. (**A**) Heat-map of the log2-fold change values of DEMs after 1 h, 8 h, and 24 h of MeJA treatment compared to the control. DEMs were divided into four different clusters (indicated by color-coded bars with numbers) based on their expression patterns. Columns denote the time points (1 h, 8 h, and 24 h) of MeJA treatment. The scale of heat-map is given as log2 fold change with a range from −1.0 (yellow) to +1.0 (red). (**B**) Box plots represent the log2 fold change (MeJA-treatment/control) in gene expression after MeJA treatment in four different clusters. Each single dot represents a miRNA.

Venn-diagram analysis revealed that 17 DEMs were common in all three time-points, whereas 16, 14, and 4 DEMS were common in 1 h–8 h, 8 h–24 h, and 1 h–24 h comparisons, respectively (Fig. 3B and Supplementary Table S9). Among the 17 common DEMs, 7 miRNAs (cro-novel-73, cro-novel-92, cro-novel-62, cro-novel-10, cro-miR393d, cro-miR164a, and cro-miR319a-5p) were upregulated and 5 miRNAs (cro-miR319a-3p.2-3p, cro-miR319a, cro-miR398f, cro-novel-64, and cro-novel-130) were downregulated at all three time points (Fig. 3B). In addition, the expression of five miRNAs (cro-novel-71, cro-novel-38, cro-novel-98, cro-miR168a, and cro-miR160h) were down 1 h following MeJA treatment, but were up after 8 h and 24 h. The DEMs were divided into four groups according to their expression patterns (Fig. 4A). The mean expression of DEMs in group-I (n = 30) increased after MeJA-treatment, peaking around 8 h. Those in group-II (n = 21) were initially upregulated by MeJA but gradually downregulated from 8 h to 24 h. The members of group-III (n = 17) were downregulated in response to MeJA; however, some members, including cro-novel-2, cro-miR396, and cro-miR396a-3p-4, were upregulated significantly between 8 h and 24 h. The mean expression of DEMs in group-IV (n = 21) increased continuously for 24 h (Fig. 4B).

### Identification and classification of cro-miRNA targets

In order to explore the roles of DEMs in diverse biological processes, their putative targets were predicted using the open source web server, psRNATarget^48^, with default parameters. The transcript sequences of the *C. roseus* genome were used as a reference set. Detailed information related to the predicted targets for conserved and *C. roseus*-specific miRNAs were included in Supplementary Table S10. A total of 519 candidate target genes were identified for 80 cro-miRNAs (149 target genes for 33 conserved miRNA and 374 target genes for 47 novel miRNAs, respectively), with an average of 6 targets per miRNA. The majority of the target genes (65.3%) were predicted to be regulated by transcript degradation, whereas the remaining targets are regulated by translational repression. Furthermore, 55 genes were targeted by more than one cro-miRNA. Among the conserved miRNA targets, many encode TFs, such as MYB TFs (targeted by cro-miR159), NAC domain-containing proteins (targeted by cro-miR164), and auxin response factors (regulated by cro-miR160). In addition, genes encoding receptor-like protein kinase, pentatricopeptide repeat (PPR) and tetratricopeptide repeat (TPR)-like superfamily proteins, and major facilitator superfamily proteins, were also identified as potential targets of cro-miR396 and cro-miR398. Potential targets were also identified for 47 *C. roseus*-specific miRNAs. These targets included mRNA encoding receptor-like protein kinase, ubiquitin protein ligase 6, blue-copper-binding protein, sterol methyltransferase 2, and F-box family protein. In addition, some *C. roseus*-specific miRNAs were also found to target TFs, including basic bHLH, bZIP domain proteins, and ARF family TFs (Supplementary Table S10).

**Figure 5:**
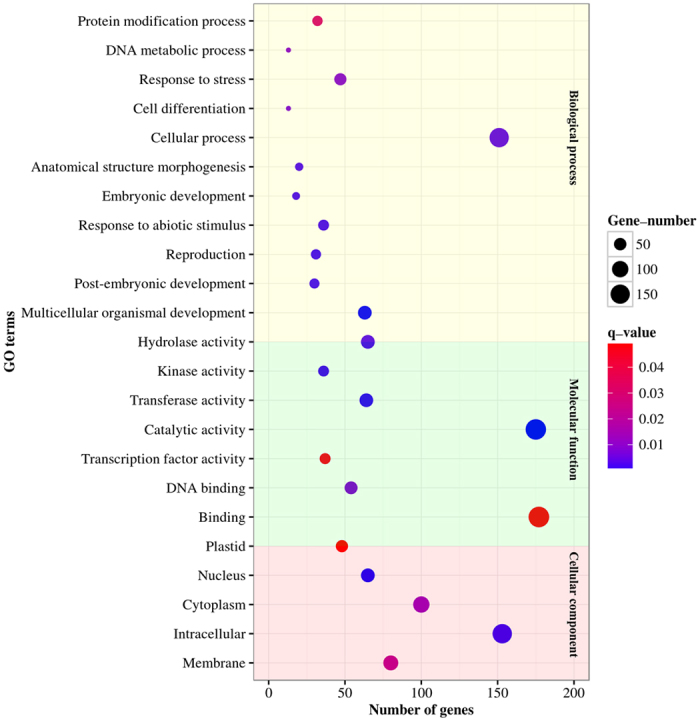
Gene ontology (GO) analysis of target genes of DEMs in *C. roseus* seedlings. Each GO term is represented by a single circle, that the color indicates the q-values and the significance of the GO term, and the size is proportional to the gene numbers. The Y-axis represents the names of enrichment GO terms. The X-axis represents the number of genes.

The potential targets of the DEMs were annotated to better understand the biological functions of cro-miRNAs. Gene ontology (GO) analysis revealed that these target genes could be classified into 11 biological processes, 8 molecular functions, and 5 cellular components (Fig. 5). For biological processes, “cellular process”, “multicellular organism development”, and “response to stress”, were the three most dominant GO categories. With regard to cellular components, “intracellular’ and ‘membrane” were the two most abundant GO terms. The GO term “intracellular” was significantly enriched for two children GO terms, “nucleus” and “cytoplasm”. The three most dominant GO terms in molecular functions were “binding”, “catalytic activity”, and “transferase activity”. Analysis of the GO term ‘transcription factor activity’ revealed that several families of TFs, such as GRAS, MYB, ARF, NAC, and GRF, were more dominant in this category. A significant number of genes associated with ‘kinase activity’ (mostly receptor-like kinases) were also found to be targeted by the cro-miRNAs. The GO term “catalytic activity” was enriched with children GO terms, “transferase activity” and “hydrolase activity”.

**Figure 6:**
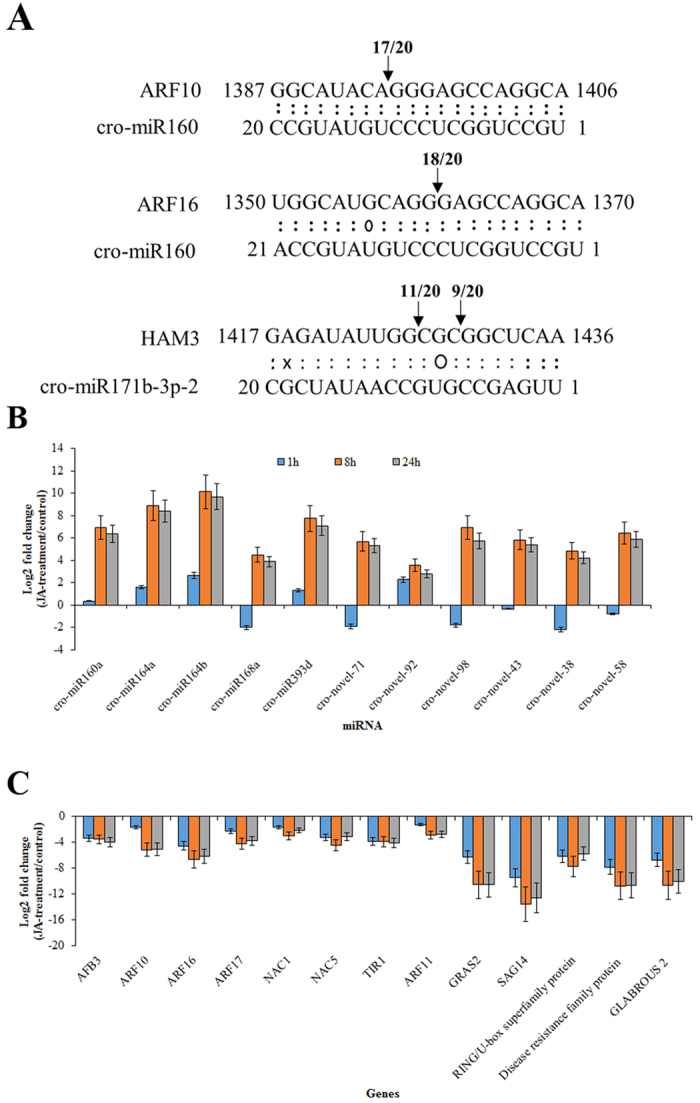
PPM-RACE and experimental validation of selected conserved and novel cro-miRNAs, as well as their predicted targets. **(A)** PPM-RACE confirmed the cleavage of *CrARF10* (CRO_T007962) and *CrARF16* (CRO_T023928) by cro-miR160 and that of HAM3 (CRO_T018940) by cro-miR171b-3p-2. cro-miR171b-3p-2 cleaves *HAM3* at two sites. The numbers above the sequences indicate the numbers of cleaved fragments detected at the cro-miR160 or cro-novel-43 target sites relative to the total fragments sequenced. For example, of 20 sequenced PCR fragments of ARF10, 17 were cleaved at position 1394 by cro-miR160. Watson-Crick pairing (:), G–U wobbles (O), and mismatched base pairing (X) are indicated. **(B,C)** Quantitative RT-PCR validated the expression of selected conserved and *C. roseus*-specific miRNAs (**B**) and their targets (**C**). The expression values of miRNAs and their targets were normalized against the expression of the endogenous controls, U6 snRNA and RPS9, respectively. The log2-transformed fold changes in mRNA levels (MeJA-treated/control) of miRNAs and their targets is shown. The data represent the mean values ± SD of three biological replicates.

Previous studies suggest that auxin negatively affects the expression of key TIA pathway genes in *Catharanthus*^18^,^19^. However, the underlying molecular mechanism of IAA-mediated repression is not well studied. ARFs are key components in auxin signaling pathway. Our target prediction analyses revealed *CrARF10, CrARF16,* and *CrARF17* as potential targets of MeJA-induced miR160. *HAM3*, a GRAS domain TF, was also found to have a high target prediction score in our analysis. We performed PPM-RACE on three selected targets, *ARF10, ARF16*, and HAM3 to demonstrate that they are indeed targeted by the respective miRNAs. Cloning of the PCR products, followed by sequencing of the PPM-RACE products, confirmed that *ARF10* and *ARF16* were cleaved by the conserved cro-miR160, whereas the GRAS domain TF HAM3 was cleaved by cro-miR171b-3p-2, in *C. roseus* cells (Fig. 6A).

### Expression profiles of DEMs and their targets

We used qRT-PCR to validate the small RNA sequencing (sRNA-seq) results of the DEMs and their potential targets. Expression of eleven (5 conserved and 6 novel) DEMs (Fig. 6B) and thirteen corresponding targets were measured using qRT-PCR (Fig. 6C). Expression of *cro-miR393d, cro-miR164a*, and *cro-miR164b* were upregulated, and that of cro-miR168a was downregulated, following 1 h MeJA treatment. There was no significant change in the expression of *miR160a* in response to 1 h MeJA treatment. The expression of all the selected conserved miRNAs were induced following 8 h and 24 h MeJA treatments. All analyzed *C. roseus*-specific miRNA were upregulated after 8 h and 24 h JA treatments. These results are in good agreement with the sRNA-seq data.

Usually, miRNAs and their target genes are expected to have contrasting expression patterns. We thus analyzed the expression of a number of TF genes (*ARF10, ARF16, ARF17, TIR1, AFB3, NAC1, NAC5* and *GRAS2*) which were predicted to be targeted by conserved and novel miRNAs, including cro-miR160, cro-miR3953d, cro-miR164, cro-novel-43, and cro-novel-38. As expected, the expression of the conserved and novel cro-miRNAs were inversely correlated to those of their targets (Fig. 6C).

### Negative regulation of TIA structural and regulatory genes by auxin

TIA biosynthesis is influenced by a number of phytohormones including auxin and MeJA. Our results showed that expression of *CrARF10, CrARF16*, and *CrARF17* were repressed in MeJA-treated *C. roseus* seedlings. In addition, these three ARFs were identified as targets of MeJA-induced miRNA cro-miR160. The expression of *TIR1* and *AFB3*, crucial components in auxin signaling, were also altered in the MeJA-treated seedlings.

**Figure 7:**
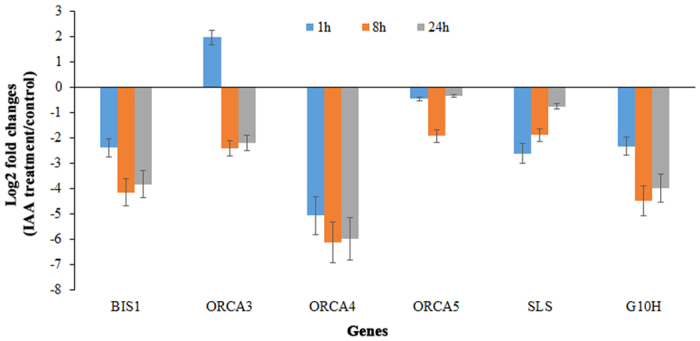
Quantitative RT-PCR analysis of selected TIA biosynthetic pathway genes in control and IAA-treated *C. roseus* seedlings. The relative abundance of individual gene is presented as the ratio of the IAA-treated vs. control seedlings. The expression value of each gene was normalized against the expression of endogenous control, RPS9. The log2-transformed fold changes in mRNA levels (IAA-treated/control) of the target genes is shown. The data represent the mean values ± SD of two biological replicates.

Auxin has been shown to represses the expression of several TIA pathway genes, including *TDC* and *STR*, in *C. roseus* cell lines^18^,^19^. Previous findings, and our analysis of JA-treated *C. roseus* seedlings, confirmed that most TIA biosynthetic pathway genes are induced by exogenous application of JA (Supplementary Fig. S4). We thus hypothesized that JA and auxin act antagonistically to regulate the TIA pathway in *C. roseus.* To test our hypothesis, we analyzed the expression of key regulatory and structural genes in *C. roseus* seedlings treated with IAA for different time periods. As shown in Fig. 7, auxin repressed the expression of key regulatory (*ORCA3, ORCA4, ORCA5, BIS1*) and structural genes (*STR, TDC, G10H, SLS*) in the TIA pathway. The transcript levels of *BIS1, ORCA4, SLS,* and *G10H* were significantly decreased at all time-points following IAA treatment. The expression of *ORCA3* was not affected by IAA at 1 h, but reduced after a longer treatment. The expression of *TDC, SGD,* and *STR,* were moderately affected by IAA treatment (Supplementary Fig. S5). In addition, we also measured the expression of two ARFs, *CrARF10* and *CrARF16,* in IAA-treated *Catharanthus* seedlings using qPCR. Expression of *CrARF10* and *CrARF16* were induced significantly following IAA treatment (Supplementary Fig. 6).

**Figure 8:**
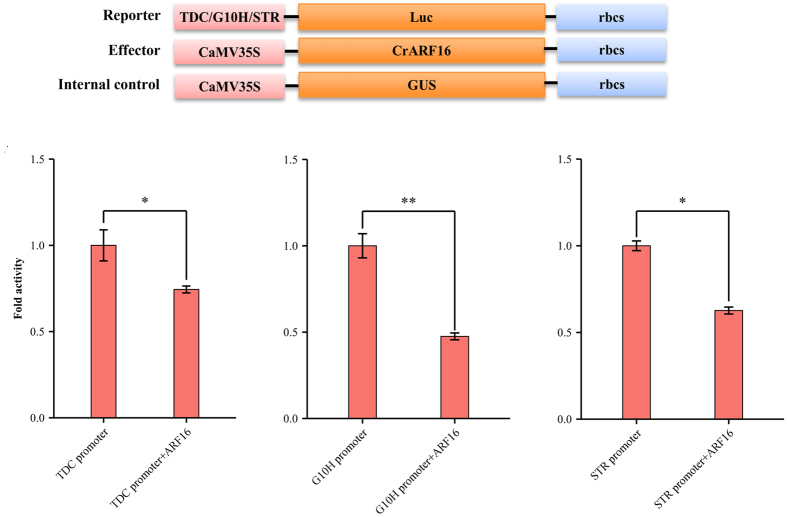
Repression of *TDC, G10H,* and *STR* promoter activities in tobacco cells by CrARF16. *TDC, G10H,* and *STR* promoters fused to the *fire-fly* luciferase reporter were electroporated into tobacco protoplasts either alone or with the effector plasmid expressing *CrARF16*. A plasmid containing the *ß-glucuronidase (GUS*) reporter, controlled by the *CaMV*35S promoter and *rbcS* terminator, was used as a normalization control. Schematic diagrams of the reporter, effector, and internal control plasmids used in this assay are shown on the top. Luciferase and GUS activities were measured 20 h after electroporation. Luciferase activity was normalized against the GUS activity. The reporter alone without effectors served as the control. Data presented are means ± SDs of three biological replicates. Asterisks indicate a significant difference compared to the control at *P < 0.08 or **P < 0.005 (t-test).

The repression of TIA pathway genes by IAA led us to hypothesize that ARFs repress TIA pathway genes by binding to key pathway gene promoters. To test our hypothesis, the promoter sequences of *TDC, G10H,* and *STR* were PCR-amplified, from *Catharanthus* genomic DNA using gene-specific primers, and cloned into a plant expression vector containing a firefly luciferase reporter^9^; the promoter-driven transcriptional activities were measured, alone or in the presence of CrARF16, in a tobacco protoplast assay. As shown in Fig. 8, all three promoters individually generated significant background luciferase activity. However, co-electroporation of the promoters-reporter plasmids with CrARF16 significantly repressed the basal activities of all three promoters.

## Discussion

Here, we report the identification, expression analysis, target prediction and validation of conserved and novel miRNAs in *C. roseus* seedlings. Additionally, we identified a set of JA-responsive cro-miRNAs and attempted to explore their potential roles in posttranscriptional control of TIA biosynthesis.

### The miRNAome of *C. roseus*

Using high-throughput sequencing, we identified 354 cro-miRNAs (Supplementary Tables S2, S3, S6 and S7), a number that is close to what have been reported from *Arabidopsis* (427), *Zea mays* (321), *Sorghum bicolor* (241), and *Populus trichocarpa* (401), but less than those from *Medicago truncatula* (756), *Oryza sativa* (713), and *Glycine max* (639). A previous study has identified 81 conserved miRNAs in *Catharanthus* seedlings^40^. Most of the conserved miRNA family members identified in that study are present in our dataset. The 24 nt long sRNAs dominated the *C. roseus* sRNA transcriptome, indicating the dominance of heterochromatic small interfering RNA (siRNA) (Fig. 1A,B). Similar observations have been made for many other plant species, including peanut, hot pepper, cucumber, rice and trifoliate orange^49^,^50^,^51^,^52^,^53^. The high percentage of 24 nt sRNAs probably plays a vital role in preserving genome integrity by heterochromatic-histone modification^54^. Our dataset also shows a high ratio of total reads/unique reads for the 21 nt class. The majority of the identified cro-miRNAs were 21 nt in length, which is the canonical size for miRNAs generated from DCL1 processing^55^. Based on the recently published draft *C. roseus* genome, which is not fully assembled and annotated, we were able to predict 65 precursor sequences representing 28 conserved miRNAs in *C. roseus* genome (Supplementary Table S3). Cro-miR159, -miR396, -miR166, -miR162, and -miR319 are highly abundant in *C. roseus* (Supplementary Table S4). MiR159 and miR319 are conserved family of miRNA and important for plant growth, morphogenesis, and reproduction, by regulating the expression of a number of MYB and TCP family TF genes^56^. The conserved miR396 regulates the GROWTH-REGULATING FACTOR (GRF) family TFs, which control cell proliferation in *Arabidopsis* leaves^57^. MiR162 targets DICER-LIKE1 (DCL1), which catalyzes the formation of miRNA sequences, thus controlling miRNA biosynthesis by negative feedback regulation^58^. MiR166 and miR159 are involved in abiotic stress responses such as cold and salinity^59^,^60^. A previous study has identified conserved miRNAs from 99 different tissues in 34 different plant species^61^. We show that the *C. roseus* miRNAome harbors most of the ancient miRNA families, including miR160, miR159, and miR164 (Fig. 2A, Supplementary Table S2). In addition to the conserved miRNAs, all sequenced and analyzed plant genomes contain family- or species-specific miRNAs that may have originated and diverged on scales ranging from family to species^62^. The *C. roseus* genome harbors numerous miRNAs that lack close orthologs in other plant species (Supplementary Table S7). Consistent with previous observations^63^,^64^, we also found that the majority of the *C. roseus*-specific miRNAs show relatively low expression when compared to the conserved miRNAs (Supplementary Table S8). A previous study^40^ has identified a limited number of cro-miRNAs that is significantly lower than the average numbers of miRNAs reported in other plant species. The miRNAome reported here provides a comprehensive account of the numbers and expression profiles of conserved and novel miRNAs in *C. roseus* seedlings. Unlike the previous report, the majority of the cro-miRNAs identified in the study are mapped to the *C. roseus* draft genome. Moreover, expression analysis of the selected conserved and novel cro-miRNAs by qRT-PCR complements the sRNA-seq data and validates our miRNA prediction criteria (Fig. 6A–C).

### JA-responsive miRNAs target key regulators in auxin signaling

JA-responsive expression of regulatory and structural genes is a hallmark of TIA pathway. Through modulating the expression of the TIA pathway genes, exogenous application of JA increases the production of TIAs in *C. roseus* seedlings, hairy roots, and cell cultures^65^,^66^,^67^. A number of JA-responsive regulatory genes have been isolated and characterized for their roles in regulation of the TIA pathway. However, the influence of JA on miRNA expression is not well studied. In *Taxus chinensis,* MeJA downregulates *miR156, miR168, miR169, miR172, miR172, miR396, miR480*, and *miR1310,* but upregulates *miR164* and *miR390*^68^. Our analysis shows that a number of cro-miRNAs were also differentially expressed in response to MeJA treatment (Fig. 3A,B; Supplementary Table S9) and the DEMs were divided into four independent clusters based on their expression (Fig. 4A,B). We observed a significant induction of *cro-miR160, cro-miR168*, and *cro-miR393* after JA treatment. Target prediction of MeJA-responsive DEMs identified 519 potential targets (Supplementary Table S10). GO analysis led to a better understanding of their biological functions (Fig. 5). Detailed analysis of the GO term, “transcription factor activity” revealed the significance of several auxin signaling genes, including *TIR1, CrARF10, CrARF16*, and *CrARF17*.

ARFs are a plant-specific TF family that controls auxin-regulated transcription. Most ARFs have a conserved N-terminal DNA binding domain, C-terminal dimerization domain, and a non-conserved middle region that confers transcriptional repression or activation^69^,^70^. ARFs bind to AuxREs, which are found in the promoters of early auxin response genes, including *Aux/IAA, SMALL AUXIN UPREGULATED RNA (SAUR*), and *GH3*, to either enhance or repress their transcription^71^,^72^. In *Arabidopsis, ARF10, ARF16*, and *ARF17* are reported to be targeted by miR160^73^. We observed a significant upregulation in *cro-miR160* after MeJA treatment (Fig. 6B). As expected, expression of *CrARF10, CrARF16*, and *CrARF17* were significantly downregulated after MeJA-treatment in *C. roseus* seedlings (Fig. 6C). Other than *cro-miR160*, MeJA also induced the expression of *cro-miR164a, cro-miR164b, cro-miR393d,* and *cro-novel-92,* which are predicted to target the auxin signaling genes, including *NAC1, NAC5, TIR1, AFB3,* and *ARF11.* The JA-responsive differential expression and targeting of IAA signaling genes by these MeJA-induced cro-miRNAs suggest that they can potentially affect TIA biosynthesis through regulating key factors in IAA signaling.

### Cro-miRNA-targeted ARFs are potentially involved in regulation of the TIA pathway

Previous studies have reported the negative effects of auxin on expression of several structural genes in the TIA pathway^18^,^19^. However, the molecular mechanism underlying auxin-mediated repression of TIA pathway genes is still elusive. Our results not only confirmed the previous findings, but also showed that auxin significantly downregulates the expression of several key transcriptional regulators, including *ORCA3, ORCA4*, and *BIS1*, in TIA biosynthetic pathway (Fig. 7). In addition, we showed that auxin upregulates the expression of two ARFs, *CrARF10* and *CrARF16*, in *Catharanthus* seedlings. We also demonstrated that the miRNA-targeted CrARF16 represses the activity of key TIA pathway gene promoters, such as *TDC, STR,* and *G10H* in plant cells (Fig. 8). Taken together our findings suggest that auxin and JA act antagonistically to regulate the expression of TIA pathway genes, in part, through the posttranscriptional regulation by cro-miRNAs. Auxin represses the expression of key TIA pathway genes, possibly mediated by the CrARF repressors. MeJA-induced expression of cro-miRNAs result in the degradation of CrARF repressors, leading to activation of TIA pathway genes.

In conclusion, this study provides a comprehensive account of the *C. roseus* miRNAome and the possible roles of cro-miRNAs in regulating the biosynthesis of TIAs. In addition, targeting of CrARFs by MeJA-induced cro-miRNAs to attenuate the repression of auxin highlights antagonistic functions of two key phytohormones in TIA pathway regulation. Our findings provide a starting point for further investigation of the regulatory roles miRNAs in specialized metabolite biosynthesis, in particular, TIAs in *C. roseus*.

## Materials and Methods

### Plant Materials, treatments, and RNA isolation

*Catharanthus roseus* (L.) G. Don cv. ‘Cooler Apricot’ seeds were surface-sterilized and germinated on half-strength Murashige and Skoog (MS) basal medium. Two week-old seedlings were treated with 100 μM MeJA or 10 μM indole acetic acid (IAA) for 1 h, 8 h, or 24 h and frozen immediately in liquid nitrogen. Mock-treated seedlings were used as controls. Total RNA were isolated from 100 mg of control and MeJA-treated seedlings using miRVana miRNA isolation Kit with phenol (ThermoFisher Scientific, USA). RNA quantity was determined using a NanoDrop ND-1000 spectrophotometer (NanoDrop Technologies, Wilmington, DE, USA). Quality of the RNA samples was determined using an Agilent 2100 Bioanalyzer (Agilent Technologies, Palo Alto, CA, USA). RNA samples with RNA integrity number (RIN) above 8 were used for library preparation. For expression analysis of regulatory and structural genes in the TIA pathway, RNA were isolated from the control and MeJA- or IAA-treated seedlings using RNeasy plant mini kit (QIAGEN, USA).

### High-throughput sequencing of small RNAs

Two micrograms of RNA from each sample was sent to the Sequencing and Genotyping Center at the Delaware Biotechnology Institute at the University of Delaware for small RNA library preparation and sequencing. The libraries were pooled together and sequenced on an Illumina HiSeq 2500. Deep sequencing was performed in triplicates for each treatment for a 50 cycle single end run. The data quality was checked at the Sequencing and Genotyping Center and sequencing reads were provided in the FASTq format.

### Analysis of small RNA sequencing data

Low quality and contaminated reads were removed as described previously^74^. Sequences smaller than 18 nucleotide (nt) and larger than 24 nt were also removed. Reads ranging from 18 to 24 nt in length, were mapped to the *C. roseus* reference genome sequence^41^ with the software, Bowtie2^75^, with no mismatches allowed. The unmapped sequences were removed from analysis. Reads, matched with the *C. roseus* genome, were compared with non-coding sRNAs deposited in NCBI GenBank and RNA family (Rfam) databases. Reads that matched ribosomal RNAs (rRNAs), transfer RNAs (tRNAs), small nucleolar RNAs (snoRNAs), and small nuclear RNAs (snRNAs), were filtered and removed. The remaining reads were used for downstream analyses.

### Identification of conserved and *C. roseus*-specific (novel) miRNAs

For conserved cro-miRNA identification, sequence reads were aligned with known miRNA sequences from other plant species deposited in miRBase 21 (http://www.mirbase.org/index.shtml)^42^,^76^ with a maximum of two mismatches. The unaligned reads were then subjected to miR-PREFeR pipeline to predict novel miRNA candidates. miR-PREFeR^45^ utilizes expression patterns of miRNAs and follows the criteria for plant miRNA annotation^46^ to accurately predict plant miRNAs from one or more small RNA-seq samples. The pipeline generates candidate regions and candidate mature sequences of each candidate region based on alignment depth of 20 reads. Predicted novel cro-miRNAs were further screened and validated in this study. The secondary structures of precursors for all conserved and novel cro-miRNA candidates were constructed using the RNAfold software^77^.

### Differential expression analysis of JA responsive cro-miRNAs

Differential expression of cro-miRNAs was calculated from read count data using DESseq2 of the R package as described^78^. Cro-miRNAs with log2 fold change differences ≥1, p-value ≤ 0.05, and false discovery rate (FDR) ≤0.1, were considered to be differentially expressed. The R package ‘gplots’ was used for heat map generation of differentially expressed cro-miRNAs. Normalized count was calculated as described previously^44^.

### Prediction and annotation of cro-miRNA target genes

The potential target prediction of all identified cro-miRNAs was conducted using the plant small RNA target server (psRNATarget; http://plantgrn.noble.org/psRNATarget/), a widely used web-based tool^48^. Candidate targets were analyzed by locally installed BLASTX search against the NCBI Nr database with the default parameters. BiNGO 3.0.3 plug-in^79^ of Cytoscape^80^ was used for gene ontology (GO) analysis of target genes of JA responsive cro-miRNAs.

### Quantitative RT-PCR validation of selected, differentially expressed cro-miRNAs and their potential targets

Expression of cro-miRNAs were analyzed using the poly(T) adaptor RT-PCR method^81^. One μg of RNase-free DNase I treated total RNA was polyadenylated at 37 °C for 60 min in a 10 μl reaction volume containing 0.08 units poly (A) polymerase. The polyadenylated RNA was then reverse transcribed in a 20 μl reaction mix with SuperScript III Reverse Transcriptase (Invitrogen, USA) and oligo (dT) adaptor, following the manufacturer’s instructions. For expression analysis of regulatory and structural genes in TIA pathway, synthesis of first strand cDNA from total RNA, and Quantitative RT-PCR (qRT-PCR), were performed as described previously^13^. The comparative cycle threshold (Ct) method (bulletin no. 2; Applied Biosystems, http://www.appliedbiosystems.com) was used to measure the transcript levels. U6 small nuclear ribonucleic acid (snRNA) and Ribosomal Protein S9 (RPS9), were used as the normalization controls for miRNA and target mRNA, respectively. The primers used in RT-qPCR are listed in Supplementary Table S11. All experiments were performed using two biological replicates with three technical replicates. Specificity of miRNA primers was determined by cloning the PCR products into the pGEM-T Easy vector (Promega, USA), followed by sequencing.

### Poly(A) polymerase-mediated rapid amplification of cDNA ends

Poly(A) polymerase-Mediated Rapid Amplification of cDNA Ends (PPM-RACE) was conducted using the protocol described previously^82^ to map the cleavage sites of target transcripts. Briefly, total RNA (2 μg) isolated from *C. roseus* seedlings were polyadenylated with poly (A) polymerase and used for cDNA synthesis using oligodT primer with an adapter. The first strand cDNA served as template to amplify the cleaved products using adapter and gene-specific primers. The PCR products were cloned into the pGEM-easy vector and sequenced.

### Transcriptomic Analysis of MeJA-treated *C. roseus* tissues

To analyze the effect of MeJA on *C. roseus* transcriptome, publically available RNAseq data were obtained from the sequence read archive database (accession number PRJNA185483). Raw reads were cleaned and filtered as described previously^74^. Finally, cleaned reads were mapped to the *C. roseus* reference sequence^41^ with Bowtie2^75^, and Fragments Per Kilobase of transcript per Million mapped reads (FPKM) values were calculated by eXpress^83^.

### Plasmid construction, protoplast isolation and electroporation

For the protoplast assay, reporter plasmids were generated by cloning the *TDC, STR,* or *G10H* promoters into a pUC vector containing the fire-fly *luciferase* and *rbcS* terminator. The effector plasmids were generated by cloning *CrARF16* into a modified pBlueScript vector containing the cauliflower mosaic virus (CaMV) 35S promoter and *rbcS* terminator. A plasmid containing ß-glucuronidase (*GUS*) reporter, controlled by the *CaMV*35S promoter and *rbcS* terminator, was used as an internal control in the protoplast assay. The reporter plasmids were electroporated, alone or with the effector plasmid, into tobacco protoplasts. Protoplast isolation from tobacco cell suspension cultures, electroporation, and measurement of luciferase and GUS activities in protoplasts were performed as descried previously^84^.

## Additional Information

**How to cite this article**: Shen, E. M. *et al*. The miRNAome of *Catharanthus roseus*: identification, expression analysis, and potential roles of microRNAs in regulation of terpenoid indole alkaloid biosynthesis. *Sci. Rep.* **7**, 43027; doi: 10.1038/srep43027 (2017).

**Publisher's note:** Springer Nature remains neutral with regard to jurisdictional claims in published maps and institutional affiliations.

## Supplementary Material

Supplementary Information

Supplementary Data
