# Supplementary material for: The miRNAome of Catharanthus roseus: identification, expression analysis, and potential roles of microRNAs in regulation of terpenoid indole alkaloid biosynthesis
Source: Sci Rep. 2017 Feb 22;7:43027. doi: 10.1038/srep43027 (PMC5320439; doi:10.1038/srep43027)
Supplement: Supplementary Information [file srep43027-s1.pdf]

The miRNAome of *Catharanthus roseus*: identification, expression analysis, and potential roles of microRNAs in regulation of terpenoid indole alkaloid biosynthesis

Ethan M. Shen[1†], Sanjay K. Singh[†], Jayadri S. Ghosh, Barunava Patra, Priyanka Paul, Ling Yuan*, and Sitakanta Pattanaik*

Department of Plant and Soil Sciences, University of Kentucky, 1401 University Drive, Lexington, KY 40546

[1]Math, Science, and Technology Center, Paul Laurence Dunbar High School, 1600 Man o' War Boulevard, Lexington, Kentucky 40513

† These two authors contributed equally to this work.

* To whom correspondence may be addressed. E-mail: spatt2@uky.edu; lyuan3@uky.edu

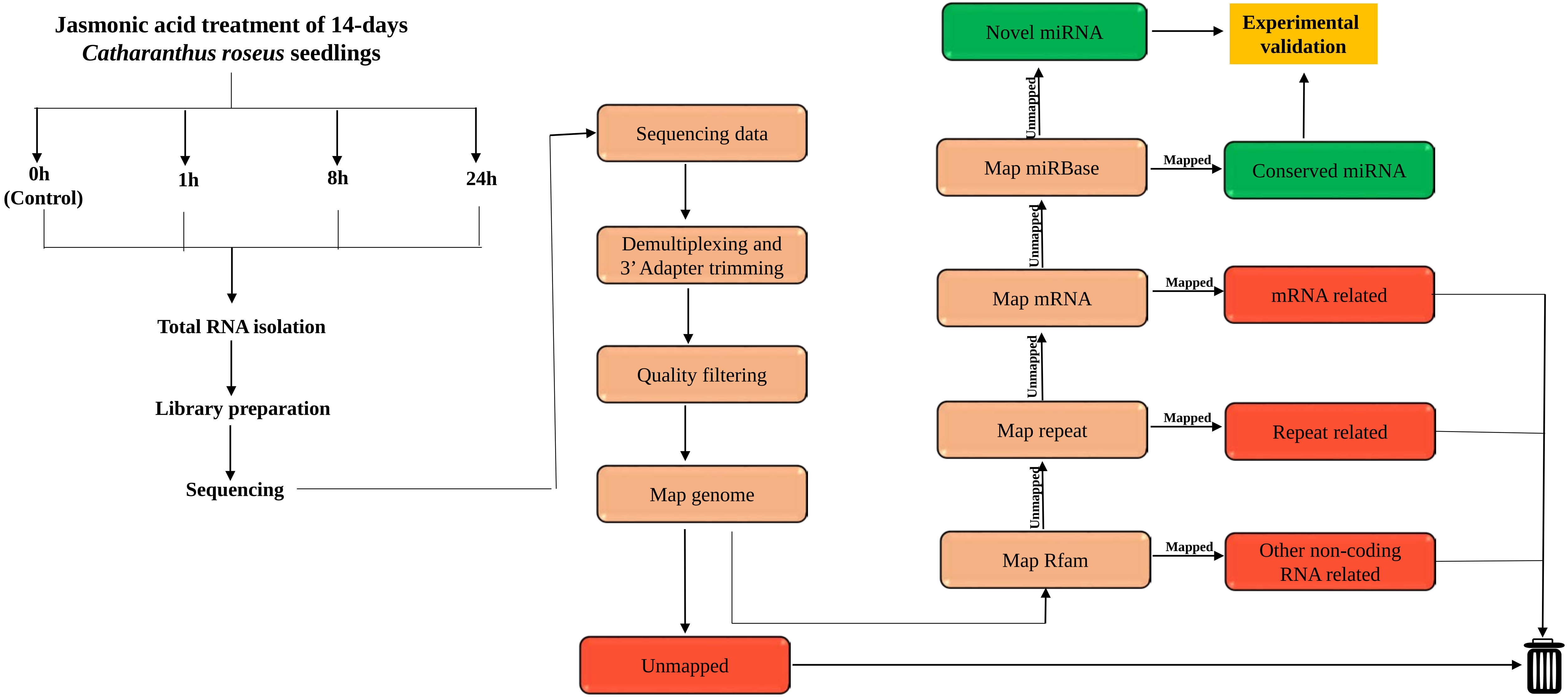

Figure S1. Schematic diagram of the experimental procedure of JA-treatment and miRNA identification in . *Catharanthus roseus* seedlings.

Figure S2. The secondary structures of the conserved cro-miRNA precursors. Mature miRNA sequences are highlighted in red.

>cro-miR1511

AUUAUAGUGAUUUGGUUUGGAUAUGUGAUUGUAUAGUUGAGAUAUUUUAGGAUAUUAUGUACCUGCUU
CCUGGUUUAUAGGAAGUUACUGCUGUACAUAUGAAAAAUGGGGGAAUAGGAAAAAGGACGAGAAAACAGU
AUAGUUUUCCUAUUGAAAACUUCAUGGUAUCAGAGCUAGGUUUCAUGGCUGUGUAGGGAGUUUAUUCGA
UACUCCAAAUAAAGUAUUCUACAUUUUUUUCUCAUUCAAACAUGGACAUGGAGGAUUAUGUUAAUGAUCA
CAUUCAAAGAGAUGCAAAUCAUUUGGU

.....((((((((((((....(((((((........(((((.....(((((((((..(((...((((((((.....))))))))...))).((((.((((((.........((((((((((...((.........))....))))))))
))......))))))))))((.(((((.......))))).))...(((((.........)))))......)))))))))........)))))......(((.((((((......)))))).))))))))))......)))).)))))))))
....

>cro-miR156-1

GUAAGAGUAGUGAGGGGCUGAUGAGGGUGAUGACAGAAGAAUAGAGAGCACAAAUGAAGGAGGUGCAGUU
GCUCUCUCCUCAUCCAUCCUUCCAACCAAUCAUGCACUUCCACAUCAUCAGUGCCCUCUAUCUUCUGUCAACA
GCUUUCACCACUCCGAUAUUAUUAUUGC

((((.(((((((.((((.((.(((((((..((((((((((.(((((.((((..((((.(((((((((((((.........................))))..))))))))))...))))..)))).)))))))))))))))...)))))
)).))))))..))))))).))))>cro-miR156-2

GAGAGAGAAAGAGAGUGGGUGGGGUGACAGAUAGAGAGUGAGCACGCAUAGCUUCAAGCAUGGGGAAUCA
UAUUAUGCAAGAAAACCACCAUGUGUGCUCACUCUCUUCUGUCACCUCCAGCUUAAUUACCCUUUU

(((((.(.((..((((..(.(((((((((((.((((((((((((((((.(.(((..((((((.........))))))..)))...)...))))))))))))))))))))))))))))))).))))..)).).)))))

>cro-miR156-3

AGUUCUAUUUGGAUUGGUAAUAAUGGUGGUGUUUAAUUUGAAAAUAAAAUAAAUUUAUAUGAUUUUUGA
GUUAAAAAAAAACUGUCCAAAUAGGGCU

(((((((((((((..........(((....((((((((((((((...(((....)))...))))))))))))))).....))))))))))))))))

>cro-miR157

AGGGCUAGAGUGAGUGAGAAAAGAAAGAAGAGGGGAGGGGGGUGAAAGUGAUGACAGAAGCAUAGAGAGC
ACAAAGGAGGUGAGAUGCAGCACACUUGAACACUUUUCUCUCUCUCAUAUGCAUUUCAUCAUCUGUGCUCU
CUAUCUUCUGUCAACACAAGUAACCUCCCUAAUUUAUUAUUGGAUUUCCACUCUUCUCCCUUCCCU

((((..((((.(((((.((((...((....(((..((((((((....(((.(((((((((.((((((((((((..((.(((((((((((........((...........)).......))))))))))).)))))))))))))))))
)))))).)))).....))))))))..)))....))...))))))))))))))))).....

>cro-miR159-1

GGAGGGUGGAGCUCCUUGAAGUCCAAAAGAGGAUUCAUAAUUUGGGCAGAUCGAGCUGCUGAGAUAUGGA
UCCCUCAGCUCUAUCCCUUACUAAGACAAAAUUUUGGAUAGGUUGUGGCUUGCAUAUAUCAGGAGCCUGCA
UUUCCCAAGUUGGAUUCUUUUUUGGAUUGAAGGGAGCUCUACACUUCU

((((((((((((((((..(((((((((((((((((..(((((((.((((((.(((.((((.(((((.(..((.((((.((((((..................))))))))).))..).))))).)))).))).)).)))))))
))))))))))))))))))))))..))))))))))))).)))))

>cro-miR160-1

UAUGCCUGGCUCCCUGUAUGCCAUUUGUAGAGCCCAUCGAAUAUCAAUGGCCUCCGUGGAUGGCGUAUGAG
GAGCCAUGCAUA

(((((.(((((((.(((((((((((((..(((.((((.((...)).)))).)))..))))))))))))).))))))).)))))

>cro-miR160-2

GGAUGUGCCUGGCUCCCUGUAUGCCACACUCAUACACCACUCUCCAUAUUUGAGGAUUUUGUGUUGCGAGU
GGCGUGCAAGGGGCCAAGCAUACC

((.(((((.(((((((.((((((((((.(.((.((((.....(((........)))....)))))).).)))))))))).))))))).)))))))

>cro-miR162-1

UAUGUAUGUAGGUGUAGAGGAUUAAGCGCUGGAUGCAGAGGUUUAUCGAUCGAUUCCUGUUCAUGCUUC
UUUUUUCUUUUUUUUGAAUUUCAGGGAACAGAUCGAUGAACCGCUGCCUCCAGUGUCCCUCCUCAACCCCC
ACCUCCACCACCAUA

......((.(((((..(((((....((((((((.((((.((((((((((((..((((((((((...................)))))....)))))..)))))))))))).)))).))))))))...)))))......))))).))...
.....

>cro-miR162-2

UAUGGUGGUGGAGGUGGGGGUUGAGGAGGGACACUGGAGGCAGCGGUUCAUCGAUCUGUUCCCUGAAAU
UCAAAAAAAAGAAAAAAGAAGCAUGAACAGGAAUCGAUCGAUAAACCUCUGCAUCCAGCGCUUAAUCCUCUA
CACCUACAUACAUA

((((...((((..((((((((((((......(.(((((.((((.((((.(((((((.(((((.((...((((...................)))))))))))).))))))).)))).)))).))))).))))))))).))))..))))
...))))

>cro-miR164-1

AGCUAGCUAGAGAUGAUGAUGAUGAUGAUGAUGGAGUGAGUAGCUCUUGUUGGAGAAGCAGGGCACGUG
CAAGUUCUUCUCAGUAUCACAUUGGAAUGGCUUCCAUGCAUGAACUGUGCACGUGCUCCCCUUCUCCAACA
UGUGUUCCUCCCUCUCCUAUACUUUCUCAAUUAAUUAUUCAUUAGCAGCU

((((.(((((.((((((..(((.((.......((((.(((.(((.(.(((((((((((..(((((((((((.........((((.(((...(((((....)))))....))))))))))))))))))..))))))))))).).))).
)))...))))........)))))....)))).)).)))))))))

>cro-miR164-2

GCCGCAGGAGUAGUGGAUUGCCCAAGCUGGAGAAGCAGGGCACAUGCUAAAUUUCUUCUUAGGCACUGAU
UUGAAGUUUUGCAUGUGCUCUUGCCCUCCAGUUUGGGUCACCCCUAUAUUUUUGGC

....((((((((..((..((((((((((((((..((((((((((((((.((((((((...((((...))))...)))))))).)))))))).))))).))))))))))))).))..))..))))))))..

>cro-miR166-1

GAGACGAUAAGUCCUGAAUUUGAUGACAUAUGUGAUGGAAAGUGAGGCGUGAGGAAUGAAGCCUGGUCCGAGAUCUCCAUUACUAUUAUACUUCAAAAAACGAUUCACAAACUAAUUACUUGGUUUGUGAAAGAUGAGAUCUUGGAUCAAACUUCAUUCCAAACACCCCUUUUCCUCCUCCUUCAACCAAAUUGUUCAUCUUGUGCUUCUCUCUC

((((.((((((.....((((((.(((.....(.((.((((((.(.((.((..(((((((((..((((((((((((((.(((.....................(((((((((((....))))))))))).))))))))))))))))..)))))))))..)).))).)))))))))....)))..))))))......))))).).))))....

>cro-miR166-2

GUUGAGGGGAAUGCUGUCUGGUUCGAGACCAUUCAUUUUGAAGCUGCUGAGAUCGCAAAUUAAUUAAAUCAGACAUUAUGGUUAAUUAGAAUGAUUUCGGACCAGGCUUCAUUCCCCCCAAC

((((.((((((((..(((((((((((((.(((((...((((...((((((..................)))).))......))))...))))).)))))))))))))..))))))))).))))

>cro-miR166-3

GUUGAGGGGAAUGUCGUCUGGCUCGAAAUCCUUCAAUGAAGAGAAAGUUCUAAUUAGUGUGAUCAGAUCGAUCUUUUGAAUGAUUUCGGACCAGGCUUCAUUCCCCUCAAC

((((((((((((..((((((.((((((((.(((((....((((..(.(((.((((...)))).))).)..)))))))))).)))))))).))))))..))))))))))))

>cro-miR167-1

UUGUUUGCCGUGUACCUAUAUCAGUUGAAGCUGCCAGCAUGAUCUAAAUUUUCCUCCUUUAAAUUUUUCCUGUUGUUGUGGAAAGAUCAGAUCAUGUGGUAGCUUCACCUGUUGUUGGUUUCACGAAAAAAUCAA

...(((..((((.(((.(((.(((.(((((((((((.((((((((...((((((........................))))))...))))))))))))))))))).))).))).)))..))))..))).....

>cro-miR167-2

AUGUGGUUGUUGAAUGGUGCCCAAAGAAGGAAGGAGUGAGGCUGUCAGAGCAUGACCUAGCUACGGUUACACCCAUUUUUAUACAUACCUAUAUUUAUAUAUAACCAUGGAUAGAUCAUGCUGGCAGCUUCACUUGUUCCCCUUCUAGACCAUAU

((((((((...(((.((...........((((.((((((((((((((..((((((.(((.(((.(((((..........((((......))))........))))).))).))).))))))))))))))))))))).)))))))))..)))))))

>cro-miR168

GUGCUUUUGCGGCGGUCUCUAAUUCGCUUGGUGCAGGUCGGGAACUGAUUUGCCUCGCGGCGUCCGAUUGAUUUUGUGUUGCACACGUAUUUCUACGUGAAAGAAUACGCAUCUGUGACGCUUCUGGUAAUUGAGCUCCCGCCUUGCAUCAACUGAAUCGGAGACCGCGGUGAAUAAC

.....(((((.(((((((((.(((((.((((((((((.(((((.((.(.(((((..(.((((((((...(((..((((((...((((((....))))))....)))))).))))).)))))).).))))).).)).))))).))))))))))).))))).))))))))).))))).....

>cro-miR171-1

GAGAGGGAUUAAUAUUUGAGAAGUAGACACGGCGUGAUACUGAUAUCGGCUCAUCAGCAUGCAGUGCAUG
UGAAGCAGACUCUGUUUUGCAAAUUGUUAGUUAAUUAGCCGAUCGAUGAUGAGCCGAAUCAAUAUCACUC
UUGUAUGCUUUUCCACCUCUU

((((((..........((((((((.(((.((.((((((.((((.((((((((((((((((...))))))(((((((...)))))))((.(((((.....))))).)).......))))))))))))))).)))))).))))).)
)))))))..))))))

>cro-miR171-2

GCAUAUCAAUAACAAUACGGGAUAUUGGUGCGGUUCAAUUAGAAAACCGUGCUCUUAUACUUAGAGCCCUG
UUUGUUUGAUUGAGCCGUGCCAAUAUCACGUAUAUAUGGUUUGC

(((.(((((.((...(((((.((((((((((((((((((((((((((((.(.(((((.......))))).).)))).))))))))))))))))))))))))).))))).)))))).)))

>cro-miR171-3

GUUUCAUGUGAAGAAAAAAAGCAAGAAAUAACGAGAUAUUGGUGCGGUUCAAUGAGAAAAUAAUGCUCGA
AAUAUUCGGAUAUUGGUUUUUGAUUGAGCCGUGCCAAUAUCGCGUAUUUGAGGCUGUUUUUGUAGACAU
GAGAU

(((((((((..(.(((((.(((...(((((.((.((((((((((((((((((((.(((((.(((((.(((((...))))).))))).))))).))))))))))))))))))))).))))))))...))).))))).)..)))))))))

>cro-miR171-4

UGUUGAAAUGUUCUCUGUGAUAUUGGCUUGAUUUACUCAGAUAUGUUAAACUGCAGUGAUCAAGAUUGG
UAAAAUUAAUAAUAUUCAAGUAUGGUUACAGUUUAAUGAACUUUGAUUGAGUCGUGCCAAUAUCCCAUAG
UCAUUUCAUUCA

...(((((((..((.((.((((((((((.(((((((.(((((...((((((((...(((((((...((((..............))))...)))))))))))))))....))))).))))))).))))))))).)).)).))))))).
...

>cro-miR171-5

GAAUGAUCAUAGCAUGAAAUGAGAACUUUGAUAUUGGCCUGGUUCACUCAGACAAACACCAUUUAUUAAAG
GGGAGUUUUGAUUUUGAUUGAGCCGUGCCAAUAUCUUAGUUUUGUUUUUCAUUUUCUUCACUUC

((((((.......((((((..((((((..(((((((((.((((((((.(((((((((((.((.(((....))).)).).))))..))))).))))))).)))))))))..))))))...))))))).....))).)))

>cro-miR172-1

AGAGAGAUUUUAGCGAGUAAUUGCCGCUGCAGCAUCAUCAAGAUUCCCAUCAUCAAAGAGCGUUGUGGGAU
UCAAACUCCUCAGAACUCUAAGAAAAAUUUGUGAAUCUUGAUGAUGCUCCACCAGCAAGAACUGGCUGAUU
AUGUUUCU

(((((.((.(((((.(((..((((.(.((.((((((((((((((((.((.........((.(((.((((((.......))).)).))).))..........)).))))))))))))))))).)).).))))..))).)))))..)).)))
))

>cro-miR172-2

GGACUUGCAUGGAAAAAGAUGAUAUUCCUUUAUACCAAGAGUCAGUGCUUGGAGAUGCAGCACCAUCAAGA
UUCACAAACCUUAUACUUAAUUAAGUCAUUCUUAAUGUUUGAGAAUCUUGAUGAUGCUGCAUCACCAAACA
UAGACUAUCAUAAAAUCACCUCGCCUUUUGAAGCACACUGUAUCGUCU

((((.((((((..(((((.(((......(((((......((((.(((.((((.(((((((((.(((((((((((.(((((..........((((((.....))))))))))).))))))))))).))))))))).)))).))).)))
)...)))))......))).)))))......)).))))..))))

>cro-miR2111

AAGUUACAAAAGAAAAGGAAGGGGAUUCAGAGAAUGCUAAUAAAGUGCAAUAAAAAAGGACAAGAAAACAU
ACUUCACCUUGUAGAGCAGUCACUAUUGAUCUUGUCCUACUUUUAGAUGGAAAGAUUGGCUGUGAGGACA
AGAUAAUCUGCAUCCGGAGGACUAGAUCAAGAUUCAAAACCAAAUGCAUGUACUGAUCUAAACCUCAGGAU
GCAGAUUACCCAAUCCUCACCCAAUAUAUAUUGUUUUUUUGUGUUUGAUGAUAAUUUGCAAGAACAAGUC
CACAAUGAUCAUAUAUAGACAAGGCCAUAUAUGAAUUUAUAGUACUUGAGAAAGCAUAUUACUAUUUCUA
UUUUCUUUUUUUUUUUUUUAUACUU

.(((...((((((((((((((((((..((((((((((....((((((.(((((..(((((((((.........(((....((((........)))))))))))))))).(((((.....)))))(((((..(((((((...(.((((
((((((((.((((..((((((((.((.((........)).)).)..))))))))..)))).))))))))))))).)...))))))))))))...((((((...(((((.((((.(((....))))))).)))))...)))))).(((((
(((.(......).))))))))).))))).))))))).....)))))........)))))))))))))))))))))))))...))).

>cro-miR2275

GAAGAUCUGUUUUUUUUUUUUUUCUGUUCUUGUCUAGUUAGUGAAUGGAGAAGUUGAUCUUUCCAUUACU
GUUCAGCACAGCAGUUGGAAAUAAGAUAUUGGAGGAAACUAAACAAACAAACUAGUGAGAGAUUUGUUUA
GCAUCCUCCAAUUCUCACAUUCAACUUGAGCUCAAAAUCUUCAUACUCAGGAGGAAGUAUUGCUGCUAUCU
CUUCUUCUUCUCUACUGUUCUAAAGGGCAGAACAGGAGAAGAUAAUCUUU

((((((.(((((((((((..((((((((((...((((.((.(((.(((((((..(((.((((((..((((((......))))))))))))..(((.(((((((((..(((((((((....((....))...)))))))))..))))
))))))))....(((..((((((...............))))))..)))(((....))).))).)))))))))).))))))).....))))))))))).)))))))))))))))))

>cro-miR319-1

AGCUAGAAAAAAAGGGAGCUUCCUUCAGUCCACUCAUGGGUGGGGAGAAGGGAUUGAAUUACCUGCCGACU
CAUUCAUUCAAACACAAUAGAAGAGAUUAAGGAUAAUACUCUAUUGGUGAAUGUGUGAAUGAUGCGGGAG
GUAAACUUCAUCCUUCCCCUUCUCUGUGCUUGGACUGAAGGGAGCUCCCUCUCUCUCUCU

....(((.....((((((((((((((((((((..((((((.((((.((((((..((((((((((.(((..((((((((.((..(((((((((.(..((((....)))).))))))).)))..)).)))))))))..))).)))))))).
.)))))))))))))).).)))))..))))))))))))))))))))).....)))....

>cro-miR319-2

AGAAGGAGCUCCUUUCGGUCCAACACCCAGGGCGGAGGAGCAGUGAGAGCUGCCAUUUCAUGCAUUGGGUU
AUGCUUUGGUGUUUUUGUGUUUCUUUGUGACAAAAAGCGGUGGGCUGCCGGAAUUGAAAUGCAAAGCUU
AACCAGUGCAUGGUGAGGGAGCAACUCCUCCUGCCAACUUCGCCCGCCCAUUGGACUGAAGGGAGCUCCUU
UU

.((((((((((((((((((((((......((((((((..((((.(((.(((.((...(((((((((((.(((.((((((...(((((((...........))))))).((((.....))))...........)))))).)))))))))
)))))...)).)))..)))...))))...))))))))).....))))))))))))))))))))).

>cro-miR319-3

GAUUAUUUGUACGGAAAUUAGAGUAGAAAGAAGAGAAAAGUGAACGAAGGGAGCUCCCUUCAGCCCAAGAC
AUGACAGAAAAAACUGAACAACAGCUCCUGCUGUUGGCCCAGUGGCCCAUUUUCACACUAUUUUCAUUCUU
UUUAUUUUUGGCCCAUUUAAUC

(((((..((...((.....((((((((((((((((((.((((...((((((....))))))..............(((......)))..(((((((....)))))))(((....)))..........)))).))))).)))))))))))))
...))))..)))))

>cro-miR319-5

GCAGUGGUUGUUAAAUAAAAAGAGAAAGAGAGCUUUCUUCAGUCCACUCACGGGGUGCAGUAGGAUUUCA
AUUAGCUGCCGACUCAUUCAUCCAAAUGCUCCGAGAAUGAAUAUGAGAAUACUCGAGGGUAACUGAGUGAA
UGAUGCGGGAGACAAAUUGAAUCUUAAGCUUCCUGUACUUGGACUGAAGGGAGCUCCCUUUUCUUUAUUA
CCUACCACCGC

((.(((((.((..(((((..((((((.(.((((((((((((((((((...((((((.((..(((((((.(((((.(((.(((..((((((((.((..(((((((((.((..........)).)))).)))))..)).))))))))..)
)).)).).)))))))))))))).)).))))))...)))))))))))))))))))).)))))))))))))..))))).))

>cro-miR390-1

GGUUUGGAGAAUCUAUGAAGCUCAGGAGGGAUAGCGCCAAUGUGAAUUUGGUCUUAUUAAUAUAUAUUC
UCCAAUUAAUUAAUGGUGCAUUAAUUCUGAUCAAUGAAUAUCCUUAGCGCUAUCCAUCCUGAGUUCCAUG
GUUUCUUCACAAUC

(((((((((((.(((((.(((((((((.(((((((((..((((....((((((..((((((.(((...................))).))))))...))))))...))))).....))))))))).))))))))).))))).))))))))
.))))

>cro-miR390-2

UGCUCCAAUCGACAUGAAAAUGCCUCCCGUGCUUGAUCUUUACACGUUAAAUUCGUUUCCUUGUAUGAAUA
UAGUAAAACCUCUUGAUUAUUUUUCUGUCAUUGCGUUCAGCAGUGGAGGAUCUGCAAAGCUCAGGAGGGA
UAGCGCCAUAGAUAAUGAAUGACCGAUGAUCAUCAUCAUUUGGUAAUUCUUCACUUCAUUUGUUGGCGCU
AUCUAUCCUGAGUUUCACGGAUCCUUCAUGCUCUUUCUCUUGGGUACGGGUUGCAGUUUCCGAUUAGCA

((((..(((((....((((.(((..(((((((((((..............((((((........))))))).......(((....((((.........))))....))).((((.(((((((((((..((((((((((.(((((((((((.
.((..((((((((..(((.((((........)))).)))..))).))))))))..))))))))))).))))))))))..)))))))))))))))).......)))))))))))..))).))))))))))))))

>cro-miR390-3

AGCAGUGGAGGAUCUGCAAAGCUCAGGAGGGAUAGCGCCAUAGAUAAUGAAUGACCGAUGAUCAUCAUCAU
UUGGUAAUUCUUCACUUCAUUUGUUGGCGCUAUCUAUCCUGAGUUUCACGGAUCCUUCAUGCU

((((.(((((((((((..((((((((((.(((((((((((..((..((((((((..(((.((((........)))).)))..))).))))))))..))))))))))).)))))))))))..)))))))))))))))

>cro-miR393

AUUUUAGUGGGAAGAUUUCUACAACUGCAACUGAAGGAGGCAUCCAAAGGGAUCGCAUUGAUCCCCAAAUU
AAUAAAUGGAAAUUAUUAGUGCAUGUAUUGUAUGGUGAAUUGGGAUCAUGCUAUCCCUUUGGAUACCUCC
UUUGGUAGCUAAAAGAUUGAUGACAUCAUCCGGCCGGAAAU

.((((.((.(((.(((.((..(((((((.(((((((((((.(((((((((((((.((((.((((((................((((....(((((.....)))))....)))))))))))))).))))))))))))).)))))))))))
.))....)).)))..)).))).))).)).))))..

>cro-miR394

CAGGAGAGGAUAGAUGUUGCAGAAAGAAUUUGGCAUUCUGUCCACCUCCGUCAAGUAUAUACCUAGCUUG
GAGGGGGCCAGCAUGUCAAAUUGGCUCUGCAUAUCUUCAUCUG

((((.(((((((.....((((((...((((((((((.((((((.((((((...(((.........)))))))))))).))).))))))))))...))))))))))))).))))

>cro-miR395-1

AUGAGAUCAGAUGUUCCCUAGAGUUCCCCUGAGCACUUCAUUGGGUUAUAUUUUAUUUAAUUAGGGCAAA
ACCCCAAACCCUAAAAUAUCCUUUAAUGAUCGGUGAUCGCCCACUGAAGUGUUUGGGGGAACUCCUGGAUU
CAUUUGAUUAUAU

....(((((((((...((..((((((((((((((((((((.(((((..............((((((............))))))............((((...))))))))).))))))))))))))))))))..))...)))))))))
....

>cro-miR395-2

UCAGGUCAUCCCUAGAGCUCCCUCAAGCACUUCAUUGAUUACUGUUUGUUUUCUUCUUUAGUAACCACUGA
AGUGUUUGGGGGAACUCCUGGGCUCAUCUGA

((((((...(((..(((.((((((((((((((((.((.((((((..............)))))).)).)))))))))))))))).)))..)))...))))))

>cro-miR395-3

GUCAGGCUUCUCCUAGAGUUCCCUUGACCACUUCACUGGGGGACAAUUGCCCCUAAUAUUAGCCCCACUGAA
GUGUUUGGGGGAACUCCGGUAGCCACCUGAC

((((((...((.((.((((((((((((.(((((((.(((((.....................))))).))))))).)))))))))))).)).))...))))))

>cro-miR395-4

AUCAGGUCAUCCCUAGAGCUCCCUCAAUCACUUCAUUGAAUACUUGUUUACUUCUUGUAUGUAUCCACUGA
AGUGUUUGGGGGAACUCCUGGGCUCAUCUGAU

(((((((...(((..(((.(((((((((.(((((((.((.((((.................)))).)).))))))).)))))))).)))..)))...)))))))

>cro-miR396-1

UUGGCUUUCUGGUAUUUUAAUGGUCCUCUUUGUAUUAUUCCACAGCUUUCUUGAACUGCAUCUGCUAGAU
CGUUUCAUUCAUGGCGGCCGCUACUGCUGCUGCGGUUGAUAUGCAAAUGCUUUUUGCGGUUCAAUAAAGC
UGUGGGAUGAUAAGGACAGGAUCAAAGGUCAA

((((((((............(((((((.(((.(((((((((((((((((.((((((((((...((.....(((.(((...((.(((((.((....)).))))).))))).)))).....))....))))))))))).)))))))))))))
)))).))).)))))))))))))))

>cro-miR396-2

AGAAGAAGUCCAGGGCCAUGUUUUUCCACAGCUUUCUUGAACUUCUGGUGAACUUAAAAAUUAGUAUUAU
AAUUUCUGUGAACUAAAGAAGCUCAAGAAAGCUGCGGGAAAACAAGGCGAAUUAUCCUGGACUGAUUGU

..((..(((((((((((.(((((((((.((((((((((((.((((((((..((....((((((......))))))..))..)))..))))).)))))))))))).))))))))).)))).......))))))))..))..

>cro-miR396-3

UUGUGGGCUAAGUCCUGAGUUGCCAUGUUUUCCCACAGCUUUCUUGAACUUCUGUAUUGGCAAUUGAAUC
CUCUAGGCCAUCACCGGAGCCAGAGCCGGGCGGAUUGGGAUCAAAAUUUCAGUGAGGAAGUUCAAGAAAG
CUGUGGAAAAGCAUGACAGGACUUGCUCAUAA

((((((((.((((((((......((((((((.(((((((((((((((((((((.((((((.((((..(((((.....((....((((........))))..))...)))))...)))).)))))).))))))))))))))))))))
).)))))))).))))))))))))))))

>cro-miR397

GGUACUAUUUAAUGAACGAAAAUGCAUAUUCAUUACCUCAUUGUUCUCCUAUGUAUUGGGGAAGUUUGUA
GUAUAAUGCUUAGGAGAAGAACAAAAAUCAAAGAGCUAAAAAAUAUCAUUGAGUGCAGCGUUGAUGAAUU
CCAAGUUCCAAGUUUUGGUAUUUGUAUUCUCACUGUGGAUUCAUCAUCGCUGCACCCAAUCAUAUUUUA
CUUCUUUGAAACCAGCUUUGAGAUGGGUAAUCGGGUAUUCUAUUUUACUUCUUUCGGUAAGUUUACU

((((..(((((.((((.(((((((.((((((((((((.((((.((((((((.((((((...............)))))).))))))))).........((((((((...((((((((.((((.((((((((.(((((((((((((((.
((((...)))).)))))............))))))))))).)))))))).)))).)))))))))..))))))))............))))))))))).)))))))..)))).....))).)))).))))).))))

>cro-miR398

AGUGAAAGUUUCCAAUAGGGUCGACAUGAGAUCACAUUUUCACGUUAUUUUUCAGAUAGAAUUCUCAAGU
GUGAAUAAAAUGUGUUCUCAGGUCGCCCCUGUAGGAAUCUCUUUACU

((((((((.((((.((((((.((((.(((((.(((((((((....((((.(((.(((......))).))).))))..))))))))).))))).)))).)))))).)))).)).))))))

>cro-miR399-1

UGUGAGUUCAAGAAUAACAGUGCAAUCCUCCUUUGGCAUGCAGGAAGGUAAUUUACAAGUAUAUUUUCAC
AUGCAUGUGUAUAUAUAAUUAAUUUUCCUUUUUGCCAAAGGAGAAUUGCCCUGCAAUUCAGUCUCGCA

((((((.....((((..(((.(((((.(((((((((((...((((((((((((.....((((((...((((....)))).)))))))))).))))))))...))))))))))).))))).)))..))))...))))))

>cro-miR399-2

GAACGGCAGAUGGUGAUCAUGUGGAGUUUUGAAUAACAGUGUAAUUCUCCUUUGGCAUGCACGGUUACAA
ACAACUUAUACUUGUACAGUACACUGUGCCAAAGGAGAAUUGCCCUGCCAUUCACUCUCACAGUCCUAGUGC
AGUUU

((((.(((..(((.(((..((((((((...((((..(((.(((((((((((((((....((((((((..((((.......))))...))).))))))))))))))))))))).)))..)))))))).))))))))).))).)))
)

>cro-miR399-3

GCACUAGGACUGUGAGAGGGAAUGGCAGGGCAAUUCUCCUUUGGCACAGUGUACUGGACAAGUAUAAGUU
GUUUGUAGCCGUGCAUGCCAAAGGAGAAUUACACUGUUAUUCAAAACUCCACAUUAUCAUCAUCUGC

(((....((.((((.(((.(((((((((.(.(((((((((((((((...(((((.(((((((((.....)))))))..)))))))))))))))))))))))).).)))))))))....)))))))...))......)))

>cro-miR399-4

AGUAGUAAGCUGAAUCAGUCAUAGGGCAACUCUCCUUUGGCAUGAUGUUAAAAUUUGCUGCAACUCAGUA
GUACCAUUUAGAUACAAUAAAAUACAUGCCAAGAGAGAGUAGCCCUAAGACUGGUUAGCUUAUGGCU

(((.(((((((((.((((((.((((((.((((((.(((((((((.((((.((((.((((((......))))))..)))).))))..........))))))))).)))))).)))))).))))))))))))))).)))

>cro-miR399-5

AGAUCAUCAAGAGGUGUAUGUUUCUAUAGUACUAAGCUAAGCAGUAUUAGGGCAGCUUUCCUCUUGGCAG
UUGAUUAUUGGAUAUUUUGAUGAAAUUUCUUGAAUAUUAUUCAUUAAAGAAAGAAAAAAAUAUUCCACUU
UUCCGCCAAAGGAGAGUUGCCCUGAAACUAUUUUAGCUUAACUUGACCUAAACAUGGACUUGCAAGCCUAG
CUGAUCU

((((((.(.((((((.(((((((....(((..(((((((((.(((.((((((((((((((((.(((((....((..(.((((((((((.......((((((((((...)))))....)))))....)))))).)))).)..)).)))
)))))))))))))))))).)))..))))))))))))......))))))).))))......)).).))))))

>cro-miR399-6

AGUGUAAUUAUCCUUUGGCACGGUUACGGACAGCUUAUACUUUUCCUGUACACUGUGCCAAAGGAGAAUU
GCCCU

...((((((.(((((((((((((((((((.((......))..))).))).)))))))))))))).))))))...

>cro-miR399-7

CAUGCAUGUGUAUAUAUAAUUAAUUUUCCUUUUUGCCAAAGGAGAAUUGCCCUGCAAUUCAGUCUCGCAC
UGCAGCUAGCUAGCGCUUCUUGAGAUCCAUCAUAAAACGUUUCUAAUCUUGCUGUACAUACGUAGUUAAG
GUUUGAUUUUAAAUCUCUGGGAAAAAUAUUCGGAUCAGUUAUAUAUAUAUAUAUAUAUG

((((.(((((((((((((..(((((.((((((((.(((.(((.(((((((...))))))).((((((....((.(((....)))))....))))))..............((.((((((((((........))).))))))).))...
....))).))).)))))).....)))..))))))))))))))))).))))

>cro-miR408

AAGAGAGAGCGAGAGAGAGAGGAUAGGAAAGGGCAGCCAGGGAAGAGGCAGUGCAUGGAAUGGAAAGGCC
ACAAACAGACAUCAUAGAAUGAAAGAAAAUGCUAGAAUGCUCCAUUUCAUGCUCUGUCUCGUCCCCGUCUG
UCCUUACCAACAUACUAUGUUUCUUCCUUACCUU

.((.(.(((.((((((.(.((....((.((((((((.(.((((.(((((((.((((((((((((............(.(((..(((.((........)).)))..))))))))))))))).))))))).)))).).)))))))).)
)......)).).)))))).))).))).

>cro-miR477

GCUCUUUUUUGUUAACUCUCCCUCAAGGGCUUCUGGCCUCAUCUUGCUAAUCUUAUUGCCUCUUUGUCUU
AAUGGAAUAAAGAGGGCUAGAAGAACUUGAGGGAGAACUGACAAAUUGC

.......(((((((..(((((((((((..(((((((((.......((..........))((((((((((....)).)))))))))))))))))..)))))))))))..)))))))....

>cro-miR5532

UAUUCACCUCUUUGAGCUUCUAUUGAAUCGAGAAAUAGGUUUGAUUGUCCAUCUUUUUGACAUAUCUAAG
GCAUUCUCCGGAUAAUUCAAAUCGAAGCAAUUAGGUAUCCAACUCGGGCCUAUAUGACAUGAGCGAUCAAA
AGAAAUACUCCACCUUUGUCAUAUAUUCCAUACAUCACACUAGAUAGUAUCAUAUUCAUGGAAUACGAUUC
ACUUUCAAGAUGCCUUGGUGGUGAAAUAGUAGACACGCGAGACUCAAAAUCUCGUGCUAAAGAGCGUGAGG
UUUGAGUUCCUUCAAGGCAUAAUACGGAGAAUGCUCAUUGAAUGAGCAAUUCAAUAAGCGAGCUCGGAUC
GAAUCGGUAUAUA

.....(((((((((((((.((((((((((((.......(((((((((...((..(((((..(((((..((......)))))))..)))))..)).))))))))).......))))((..(((((((((.(((.((.............
))...))))))))))))..))(((((((......))).))))).....((.(((..(((..((((((.(((((....))))).))))))...)))..).)).))((((((((.((((((((((....))))))))))))))))))(((..
.)))..............(((((((...)))))))))))))))...)))))))))..))...))).....

>cro-miR8016

CAACUUCAUGAUCUUGAUUCGCAUGGUCCUUUUAUUCAAAAAUAUAUUUGUUUGCAUCAUCUUUCAAAGG
AAUGAAAGUUGUUGCAUCAUUAUAUCAAAUAUAUUUUUGAAUAGAAGGCCCAUGUGUGUCAUUAUCUUGA
AACUUUG

....((((.(((..((((.(((((((.((((((((((((((((((((((((..((((.((.((((((......)))))).)).)))).........))))))))))))))))))))))))).))))))).))))..))).))))......

Figure S3. The secondary structures of the novel cro-miRNA precursors. Mature miRNA sequences are highlighted in red.

>cro-novel71

GCAAUCG**AAACUUGUUGGUUAUGUCUAG**GCGUGAAAAUUUUGAUAUCUAGACAUAAUCAACAAGUUUCGA UUGA

.((((((((((((((((((((((((((((.((.((....)).)).)))))))))))))))))))))))))))).

>cro-novel1

UCCGAGCCCAGUUUUGAAAAAAAAAUAAUUUCAACUUUUUUUACAUCUAAAUUGAUAUUCUUCUACAAACU CUACAAAUUCUUCAUACAAAUUCUACAAA**UUCUUCAUAAUGAUCUGUAUCUAC**CUCCAAUACAAGUUCAAU GUAGAGAGAAGUUAGAAAGAAAUUGUGUAGAGAGAUAAAGAGUAGGAAGGAUGAAGGAAAAUAAUGAUU UUUUUCGCGCUAUACAUUCAUAUUCUUGAGUUAGAUUUAUCGGCCCCAUAGGAAAUCUAACUCGGGAAUA UGAAUGUAUAGUGCGAAAAAAAUCAUUAUUUCUCUACUCUUUCUCUNNNNNNNNNNNNNNNNNNNN NNNNNNNNNNNNNNNNNNNNNNNNNNNNNNNNNNNNNNNNNNNNNNNNNNUCGUAUUGGAGGUAGAUAU AGAUCAUUACAAAGAAUUUGUAGAAGAAAAUAUCAAUUUAGAUGUAAAAAAGUUGAAAUAAUUUUUUUUU UAAAACUGAGCUCGGA

(((((((.((((((((((((((((((.((((((((((((((.(((((((((((((((((.((((..........................((((((((((((..((((((((((((((((((((((((((((.(((..(....)
..))).)).................(((((..((((((((..........(((.((.........))))))))))))))))))))))))))))))))))
)))))))))))))))))))))))))))))))))))...)))))))))))))))))))))))))..)))))))))))))))))))
))))))))))))))))))))))))))))).)))))))))))))))))).)))))))

>cro-novel10

AAACUUUACUUUUCACUACUUUUAUCCUUC**AACUAUUUUUGGGUCACUAAAACU**GUCAUUCCCACCAAUUU UGUGGUGGCUUAGGGUGGAGACUACCACAAAAUUGGUCGAAAGGGCAGUUUUAGCGGCCCAAAAAUGGUU GGAGGGUAAAAUUGGUGAAAAGUAAAGUUG

.(((((((((((((((.(((((((((((((((((((((((((((.(((((((((((.(((..((((((((((((((((((......))).))))))))))))))).)))..))))))))))).))))))))))))))))
))))))))).)))))))))))))))))).

>cro-novel100

UUUAAGGAUAAGAUAAUUUUAAAUGAUAUAGGAGGAUGUUAAAAUUUUUGGGCUUGGGUCUCAACACAC UCCCGAAUU**AGACUGCUCUGAUAUCAUGUUAGA**AUUUUUUGGCUUUAGG

.((((((.(((((.((((((((((((((((.((((.(.(((......((((((..((.((....)).))..))))))...)))).)))).))))))).))))))))))))).))))))).

>cro-novel101

CCUACCCUCC**AGUCUGCAGGAUUGUUGAGUCACC**AUGAUUUACAUCAUUCACUUAGAUGUUGGGCAGUCUG GUAUAGAAAGAGAAGGGUAGU

.((((((((((.((((..(((..((((((....(((((....)))))..))))))..)))..)))).))))...........))))))).

>cro-novel102

UUCGGAUCCUCUAUAACAUUUCUUAUAACACUCUCUGUAACAUUCCUAAUUUGAUAUAUUUUAUUAAUAA
AACUUACCAAAUAAAAGAUAAACUCUAAAAAAAGAAUAAACUGUAAGGAAAAAAAUAUACUAUAAAAAAAAU
GUUAUAAGAAAUAUUAAAUAAAAUGUUACAGAAGAUCUGAG

.(((((((.(((.((((((((..(.(((...(((.(((((((((.......((.((((((((..........(((((.................................)))))....)))))))).)).......))))))))))))..
..))).)..)))))))).))).))))))).

>cro-novel103

AGAGUUCAAAUUUCUCUUAUGAUAUACGUUUAUUACAUAGCUUGCUUAAUGCAAUACAUUCUUAUAUAAU
UUGAGGAUUAUUAUAUAAGACGAAUUUAAGUAGUAUUUGUGGUUCCCUUGUCAAUAGAAGAAAAAACAAC
UCA

.(((((....(((((((..(((((.......((((((....((((((((..........(((((((((((.........)))))))))))).....))))))))....))))))).....)))))..)).)))))....))))).

>cro-novel104

GCUCACCAAUGUCUGGCCUCUAGUCUGUAGAGCCGCUGAGUCACCAUGAUUUACAUCUUUCACUUGGAUGU
CAGGUAGUUUGGUGGGG

.((((((((..((((((.(((((..((.((((..(.(((((((...))))))))..)))))).))))).))))))....)))))))).

>cro-novel105

CGGUCUUGCAAACUGAAGACCAGAUCUUAAUGCGCACAAGAAACUACCUUUCACAUUGAAGAGGUUGAAAU
UUCCACACUGCCGCUGCCAAGAUUUGAACUUUGGUCCUUGUUACCA

.(((...(((((((((((..((((((((...(((((...((((..((((((((...)).))))))).....))))).....)).)))....))))))))..))))))))..)))).))).

>cro-novel106

AAGAGGUUUGAGUUCGAGUCACCUAAAGAGCAGUGUUAUGCCGUAUGCGUGCUGUUUGGGUCUUGUGCA
UUUCAAUCUCUC

.(((((((.((((.((((..(((((((.((((((((........)))).)))).))))))))))).))...))))))))).

>cro-novel107

GUGCUUCUUGAAUUUUGGCACCCUGUAUUCCACUACUUUCUAUAUCCAUUUUAUGUACAUUAAUCUUCAC
UUCCUGUAUUUGCCUGGGAGUAUUUUCUGAGUAUUGGAUUGAAGGGAGCUCUGACUUAACUGCAA

.(((...((((.((..(((.((((.((.((((.(((((...((((((.......(((((...............))))).......))).))).....))))).)))).)).)))).)))..)).))))..))).

>cro-novel108

AUUUGAGAUAUCCGGGUGCUUAUCUUUACAUAUUUGAAUUCGAUCUAUAAAAUUUUUAUAAAAUCUUACU
CUUAUUAGAAUUUUAAACCCGAAAAUUAUUCAAAAAAUAUGUUGAGUUGGUGUAUUUUGAUAGCGCUCAA
AA

.((((((.((((.(((((((((.(((.(((((((((((((((.....(((((((((.((((..........)))).))))))))))....))).....)))))...))))))).))).))).)))))).))))...)))))).

>cro-novel109

GAUUCUUUCUUCUCGAUUUGUUGUUAGAUGUUGUAUGAUAUCGAACUUUUUAGAUGUUAGGUAAUCUUU
UGCUAACUACCGACUUCUGAAAUGAAAUUUCGAGUUUAUGAAAGGGUA

.((((((((..(((((((((((.(((((.((((..(((((((..........)))))))(((((....)))))......)))).)))))))))))...)))))....)))))))).

>cro-novel11

CUGUAAUAUCCUAUAUAAUACUUUUUAUAACAUUCUUAAUAGAGAAUAUUUUAUUAAUAAAUCUAUCAAA
UAAAAAUUAAACUCUAAACAAGAAAGGUAAAAUCUAAAAGAAAAAAAUUCUAAAAAAGAGUAUUACAGGAG
AUGAUAUUACAAGGAAUGAUACAGACUAUUCUAAGUGGUUUAACCAAGUUUUCUCUCACAUAAAUCGUAAC
AUUCUCUAUAACAUCUCUUGUAACAUUCAUUUUUCUAAUAUUUUUUCUCCUUAAAGUUUUCUUUUUCC
UUUAGGAAUAUUUUUUGUUUUGUAGAUUUUAUUAGUAAAAUACUCUAUAUUAGAAAUGUUAUAAAGAGU
GUUACAUAAGAUGUUACAU

.((((((((.(((.((((((((((((((((((.((((((.(((.((((((((((((((((((.(((((((((.((...((((((..(((((((.(((((.(((.((((((((((..((((((((((.(((((((((((
(.(((.....((((((.((((((..(..((...((((...))))))..)..)))..........))).))))))..))).))))))))))))).)))).))))).))..))))))))))..)))..))))))))))))..))))))..)
).)))))))))).))))).))))))))))))))).))).))))))).)))))))))))))))))))).))).)))))))))).

>cro-novel110

UUGAGUUCUAUGUCGAGUGAGAGGCGCUGAGUUCUUAUAUAUAUCUUAACUUGAUAUGUCAAGUGAGAG
GUGUUGAGUUCAAGUUAUUUAAAGAAAGAUUUUAUGUCGUAUGUAAAACUUAU

.((((((.((((((((((((((.........(((((.((.(((.(((((((((((((.((......)).))))))))))).))).))).)))))))...)))))).))).))))).)))))).

>cro-novel111

AAGUUAUUUCUUGGGGUGAAUAGGGGUGACCUCUCACCAGGGACUGGUAGAUAGUCACGUUAGGGAGUAG
CUC

.((((((((((((.((((..(((((...))))).(((((...)))))......)))).)))))))))))).

>cro-novel112

UGACUAAAAUGCAAGGACAGAAUCAAAAGGAACAUGACAGAAUAAAAGUUGUGGUUCUGUUUAUAGCGUU
UUGUUUAUUGUUCGAUGAUUUUGGUUU

.(((((((((....((((((((.(((((.(.....((((((((..........))))))))).....).))))).)).))))))....))))))))).

>cro-novel113

UUACUUUAAGAGACGUGUAUGUGUUGAGAAUCUCAUACGGCUAGUAUUUAAGAAAUGCUUACUGCUUAUA
AAGUCUUAGGCAUCCUCCCCUCUUGAAGUAU

.((((((((((....(.((((..(((((........(((..(((((((...)))))))..))).........))))).)))).)....))))))))))).

>cro-novel114

GGUGACUCAGCGGCUCUAUAGAUUGUAGGUUAAGUACUGGUGCACACAAUGAACCACCUCCGCAUUGGAGA
UGUUGAAAUUCCCAUACUAUCUUGGUCCAACUGAGCUACG

.(((.(((((..(..(((.((((.(((((........((((.((.....)).)))).((((.....))))...........)).))).)))))))..)..))))).))).

>cro-novel115

AAUCAUGCUGUCUGUCAUGAUUCGUUUGAAUGCCUUUUGCUGCCUUUUGUCUGCUUUUUGACUGCUUUU
CCUAAAGUGAUAGAAUGCUAAGAUGAAUGAUUACUUAUGGCUCUGAUA

.((((.(((((..((.((.((((((((.............((.(((((((.(((((..((.......))..))))))))))).))..)))))))).)).))..)))))..)))).

>cro-novel116

AAAGGUGAAAUUUCUUGCCUACAGAGACCCUAUAGACUGCCCGACGUUCAAAUAAAAGAUAUAAAUAAUUA
UGACUUAACGGUCUUGCAGACUGAGGCUAAAAUUUGAUGCACACAAGGAACCACCUUC

.((((((...(((((((....((((...(((.(((.((((..((((((.......(((.(((((....))))).)))))).)))..)))).))))))......))))......))))))).)))))).

>cro-novel117

UUUCUGAAAUUGUCUGGCAAUAUAUUAUAAAAAGGAAACAAGUAGAUUCAGAUUCUAAUCUUGAAUUUUA
AGGAUCUUGUUUAUUCUAUACUGUCUUAGUCAAGUACAGAAG

.(((((...(((.((((((.((((..(((((.((((..(....((((((((((....))).)))))))...)..)))).)))))..)))).))))..)).)))...))))).

>cro-novel118

UCAUCUCCGCAAUGCCCAUUCCUGUCUAUUGAAGAAUUAUUCCAUGGUUUUGAAGAAUUAUUUAGGUGGC
UUGUUAAGGUACUUGAAGGCCUGAUGAUUACUCAUGAAGAAUGGAGAUUUAAAUGGUGC

.((((.....(((..((((((...((...(((..((((((..((.((((..........((((((((.(((...))).))))))))))))))))))))..))).)).))))))..))).....)))).

>cro-novel119

CAGUACGCAUUAAUUGUCUUGUAGCAUUCAGAGGUCAGGGACUAAAUCACAGUUGUGUGUUGCGAACAUU
UGUGUGUACUA

.(((((((((....(((.(((((((((.(((..((.............))..))).))))))))))))...))))))))).

>cro-novel12

UGGAGAGAGGCUGAAUCUGCUGAAGCAGAUAUGGAAUGGCUCGGAAUCUUGCCAUUUCACUUCCUUUUUC
UCCAUUGUAGCCAAGGAUUUUUGCCGCGGAAUAGGAAGAAAGAUUAAUCCUGCUGUACAUGUGGUUUUGU
UAUUAUCUUUAUUUGUUUGUUUUGGCAGAAAUCCUUGGCUAACCAAGGGGAAAGGAAUUGAGAUGACAAA
GUUUCUUCAGGGUCUCAAUUUUUGAAUUUGAUUCAGCACUUUCAACUUCUCUCCC

.(((((((((.((((..(((((((.(((((..((((((((((((((.(((.(((((((.((((((..(((((.((((((((((((((((((((((..(((((((...(((((((((..((((.......))))..))).....))
)))).....))))))))))))))))))))))))))..)))))))))))))).))))))).))).)))))....)))))...)))))...))))).)))))))..)))).))))))))).

>cro-novel120

ACAUGUAGUAUGUAUUAAACCGCGUUACCUCUAUUAUCGAUUAUAGAAACAAUAACAGCAAGGAGUGUGA
UGUGUCUUAGUAUAGGUCACAUGA

.(((((....((((((((..((((((((((((............................)))).))))))))..))))))))....))))).

>cro-novel121

UUGUGUGAUUCGAACUCAAAAUCUUUCACUUGAUAGCUAAAUGAAAGUAUUUAAGUAAGGAGAUCGCGUA
AUUGGAUUACACAC

.((((((((((((((.(....(((((.((((((..(((.......)))..)))))))))))....).))..)))))))))))))).

>cro-novel122

CGAUGUAGUUGCUAUUUUAAAAAUAAUUUUCGAACGGCUGGUUAGUCUCUAUUUCGCACAAACUUGUAAA
AAAUAAUGAUCGACUUAGUAUUCGGUUGCAACUCGGUCA

.(((..((((((.................(((((..((((((((.(((..(((((...(((....)))...)))))..))).))).)))).)))))..))))))..))).

>cro-novel123

GGUGGUGUUACUUUGAUGAUAUUAGUGCAACGAAUUAGUUUGAUAUGUUCACAUAUAAACAGGAAAGUC
UGAACAUGAAAAAGUGUAUUUGAAGCGAUUGAUAGUAGUAAAAACAAAAUUAUCCACA

.((((((((..((((....((((((((((..((((((((.(((..(((((((((.((............)).)))))))))..))).)).)))))..)).))))))))....)))))))).......)))).

>cro-novel124

CUAUAUCAUCUUGUGUACAACUCUCUGUAUCAUUUUUUAAUUUAAUAUAUAUUUUAUUAAUAAAACCCAU
UACAAAAAAUUAAAAUCUUAAGAGAAAAAGGUAAGGAAAGUAGUACAAAAAGUGAUAUAU

.((((((((.(((.(((((.(((.(((.((((.((((((..(((((.....((((((.......................)))))).))))).))))))))))).))).))).)))))..)))))))))).

>cro-novel125

AAGGUAAUCUAUAUUUAUAUAGGUGGGUCUGUCAUCUUAAUAAAAAGUGCAGGGUCUCUUUAUAAAUGU
GUAGAAACCUA

.((((..(((((((((((.(((.((.((((.((.(((......))))))))).))))).)))))..))))))).)))).

>cro-novel126

AGAACGGUCCUACCUAAGAAAUGAGAAGAAUAUUUCUUUCCCUUUUAAAAUUAAAAAAAAAAAAAACUAGU
AUAAUAUAUAUCUAUUUGAGUCGUAAAAUUAGCCGCUAGCACCAUCUCUCUAAGUCUUUCAAAAUUUUAU
AAUAUAAGAUACUUUUAUUCUUUUAAGUCUUAUUUUUUUGGCGGUACUAUGUUUA

.(((((((.((.((.((((((((((((((((((..((((.......(((((((.............(((((.((((.(((..((.....))..)))..))))...)))))......((.....))........)))))))......)))).. ....))))))......))))))))))).)).)).)))..)))).

>cro-novel127

UUCAUAUUUAGACUGUGCACAGCAUGAAAUAAGUAUCUUCCUACUGAAGAUCUUCUUGCACAGUGUGAAA
UUGGU

.(((..((((.((((((((.((........(((.((((((.....))))))))))))))))))).))))..))).

>cro-novel128

AGAGAAGUUAGAAAGAAACUGUGUAGAGAGAGAAAGAUAAGAAAAAAUAAUGAUUUUUUCUCGUUAUAC
AUUCAUAUUCUCG

.(((((..(.(((......(((((((.(((((((((((.............))))))))))).)))))))))))..))))).

>cro-novel129

UUACGAUCUUGCACCUGACAUGCUAAGUGAAAGAUUUUGAGUUCAAGUCACUUAUAGAGCAGGUUUAAUA
UCGUAU

.((((((.(((.(((((...((.((((((((..((........))...)))))))))...))))).))).)))))).

>cro-novel13

CCUUAUACGACAAUGAUAGAUAAUUUUUUGCUAAUUUAUAUCGCAUGACAUUUACAUGUCAUCUUUAUAC
UAAGGAUAAAAUCGUCUAACUAUUAAUUUAAGUCCCAAUCCAUCACUGGAAUGGGUCAAAAUGAACUUGUC
AAUGUUUUGUAUCGGCACAUAAGUCGAAUUAUUUCAAAGCAUUCCAAUUGUUGAUUAGUGUUAUAUUAG
UGGCUGGACAAUUUUAUCCUUACUAAAGAUAAUAUUUGAAUGUCAUGUAAUACAAAUUGGACAGAAAUCA
CCUAUCAUCGCCGUAUAAGA

.((((((((.(.(((((((.....((((((((((((.(((.((((((((((((.((((.(((((((...((((((((((((.(((((.((((((((.((((.((((.((((....)))).)))).).)..(((.((((((..((((
(((((..(((((......)))))......)))))))))).)))..))).)))....))).)))))))).))))).))))))))))))).))))))).)))).))))))))))))).))).))))))).))))))....))))))).).
)))))))).

>cro-novel130

AAGGUCGGAUUUGGAGCCAUAAAUUUUAUAUCUAGAGAGUUUGGGUUUGAAUCCAUAUUUACUUUAAGG
AUACGAAUCUGAAUUUGAAAAUACCCAAAAGAUCUA

.(((((...(((((.....................((((((((((((.((((.((.......)).)))).)))))))))))))........))))).))))).

>cro-novel131

AAGGUUUAAAAUAAGCUUGAAAGAAGUGUCCGGUUGAAGUUAAAUUUGUUGCACAUUAUAUGAGAGUGU
GUUAAAGUAACUGAUGAUCUUCUUGAACGUAAUUUAAACUUA

.(((((((((....((.(..((((((.(((((((((.............(((((((.......))))))).....))))))..)))))))))..).))..))))))))).

>cro-novel132

CGAGUAUUGUCAGCGUUCAUGGAUGCUCUGAAAUAAAAAUUGUCAAGUUCUAUUUUAAUAUACCGUGAUA
AUGCUGUAACACUUU

.((((.(((.((((((((((((((...(((((((..((((....)))).))))))).))).))))))..))))))))).)))).

>cro-novel133

AUUGAUUAUAACUAGGAUACUCUGUAUUAUACUCUGAUUAUAGCAGUAUUCUUUAUUAGAGGUUGGUUA
GAAUCCUAUAUUGAUUAGG

.(((((((....((((((..((((.((((..(((((((................)))))))..))))))))))))))...))))))).

>cro-novel134

AAGAAAAUCGGAUCGGACCGACCAUCAUGACUUAGAAUCGAACUUUAGACCGAUCUGAUUUUCUC

.((((((((((((((.((((...((........)).))).......).))))))))))))))).

>cro-novel135

UGAAGAAUUCUUAAGGGACCGGCAUAUUCAGGUCCAAUACGUUGCUGUAUCUCUUAAGAUAUUUCUUUU

.((((((.((((((((((.(((((..................)))))..))))))))))...)))))).

>cro-novel136

AUAUGGGAUUGUCCACACCCCCAACUCCAGGCAUGGACAUCUGAAGCGUGAAGUUUGUAGGACUGGACAUC
UAUGA

.((((((..((((((..((..(((((.((.((.............)).)).)).)))..))..)))))))))))).

>cro-novel137

AGCCCUCUUUGUGUCUUUCUGUAGGGUUAACUUCUGUUAUUUUUUUCAGUCUGAUAGUGAGAUAUCUAC
UGAGCGGCA

.((((((...(((((((.(((((((.(((((....))))........).))).)))).))))))).....))).))).

>cro-novel138

UGGUUUAUAUCUUCCUUCAUACACUUAUUACACCCUGUUAUUGAUUAUAAAGAGAAGAACAUGAAGAGUA
AUAUGACUUAAUAUAUGCCACAUAAUCUAUGAGUGAAGACAUGAAUCU

.(((((((.(((((.(((((((((..(((((...(.((((((((..((((............))))..))))))).)...)))))..))..........)))))).))))).))))))).

>cro-novel139

CAUUGAAAUGGUGACAAGUAGGAAAUCACAUGCAUCAUUCUGUAAUGAAAUAUUACCUUGCUCGUCUUCU
AUUUCAAUU

.((((((((((.(((.((((((.(((..((((((......))).)))....))).)).)))).)))..)))))))))).

>cro-novel14

GCGGUGUAGGAGCAUAUCAUGAGAAGGAAGGCAGUGCAAUGCAGCCAAGGAUGAAUUGCCGGCGUGACGA
AUCAUGGCAAAUAGAGGCAUUAUUAUAUCCUGCGUCGGCAGUUUGUCACUUGGCUACGUUGGGCUCCCUU
CCUUUCAUGUUAUGCAACACACCGA

.((((((.(..(((((.(((((((.((((((.(((.(((((.((((((((((((((((((((((((((..(((.(((.(.......).))).)))...))..)))))))))))))))).))))))).))))).))).)))))))))
)))).)))))..))))))).

>cro-novel140

AUGCAUGCAAAAUAACGAAAGUCAAGUAGAGGACGGUUGUCAAGAAGAAAAACCAGUCUUCCUCUCGAAUU
CUGUGAGCGCGAAAUGUAA

.(((((........(((.((.((.((..((((((((((.((.....))..))))).)))))))..)).)).)).)))........))))).

>cro-novel141

GAUAGUGCAACGCGUAGAUCUAAUAGUAACAGUAUUUAUAUGACUAACUAUUUAACAAUUGUCAUAACAG
UUUGAAGGGCAAGAAAUCUCGUUAAUCUUAGAUCUAGUCUCUUAUUAUA

.((((((....((.((((((((((...((((((.((((.((((((................))))))....((((.....)))))))))).))))...)))))))))))....)))))).

>cro-novel142

AAUUUUCGGAAGCUUAGAUGAAGCUAAGAAAACACAGCGGACAUAGUUCAUACAUGGCACAAUUUAUUGCA
GUUUCUGAAGAUG

.(((((((((((((..((((((((((.........(((.......)))......))))....))))))..))))))))))))).

>cro-novel143

ACAUGAGCAUCAGAUCAAAAUAUGGAGAUAUAAAGUAAAAAUAACCUUGUAUAUAUGUAUUUUGAUCUUG
GUAACAAUACAUGC

.((((...(((((((((((((((...((((((.((.......)).))))))...)))))))))))))).)))).......)))).

>cro-novel144

CUUAACUCAUAUUAUAGUCAUGGGUAUGGAGCCAACUUCUUCACAUGCGUACUGUUGUCCAAACUUCUGAC
AUAUUGGUUGGA

.((((((.((((....((((.((((.((((..((((.................)))))))).)))).)))))))).)))))).

>cro-novel145

UUGCUCGACGUCUAGGUAGAAGAUAUAAAUCAUGGUAAUUCAGUUAUUUUCAAACUGGAGGUUAGGUAC

.((((.(((.(((((...(((((((...................)))))))...))))).))).)))).

>cro-novel146

UCAAGUAUGAACCUGACGAGUAUUCAUGGCACAAAGAGUAAGUUGUAAGUACCGUUAGACUCAUGAGUUG
C

.(((.(((((..((((((.(((((.(((((...........))))).)))))))))))..)))))..))).

>cro-novel147

AUGCAUGAUGUGAUAUCGUGACUCCCUCACUUAAAAGAGUAAGAUGUUGUGAGUUUGAGUCAUGUAC

.((((((((..((..(((((((((.(((........)))...)).)))))))..))..)))))))).

>cro-novel148

AGGGUCUGGAUUUGGAUCUAUUUCACUAUAAGGUUCUAAGUUUUGGAAUAUUCGGAGGGUUUAGAUCCA

.((((((((((((.((..(((((((..((..........))..))))))).)).))..)))))))))).

>cro-novel149

ACUUAGAAUCGUCUGUAACAUUUUCUAGAUAGUAUUAUAGAUAUUGCAUGGAAUGUUAUAGAGAAGAGAG
A

.(((....((.((((((((((..((((..(((((((...)))))))..))))))))))))))))...))).

>cro-novel15

UCAUUUUGACAAAUUAAGAAUUUGUUGUUGGAAUUCGAUAAAUUUUAUUAGGUGACAUUAUCUUCGGAU
UACUUGUAGUGCGAAGAUAAUGUUCGAAAAUGAAAUUUGAAGGUGGACAAGUAGUUCAAAAAUAAUGUUA
CAUGAUGAAAUUUGUCGGAUUUCGAUUACAAAUUUUUAAUUUGUCAAAGUGC

.((((((((((((((((((((((.((((((((((((((((((((((((.((((((((((.((.(((((((((((............((.((((((.......)))))).))..))))))))))).)).)))))))))).))))
))))))))))))))))))))).)))))))))))))))))))))).

>cro-novel150

CGCACAUUGAAUGUAGACGAAUGAAGAUUAGUCUGAAUAUAAUAUGACUUAAAAUACCACCUAUAUAUACA
CAGAUUCACUCAUAAUUCCUCAUGGUCAGACAAAAAAGUGCA

.((((.((...(((.(((..((((.((..((((((..((((.((((...............))))))))..))))))..........)).)))).)))..)))...)))))).

>cro-novel151

UUGCGGUUUCUUUUUAACUUUAAUGACGUGGGAUAUAAAAAGGGUUCAGUUUUUACUAUUCUAAUUUUG
UUCUUGUCGAGAUUGCAC

.((((((((..............((((.((((((.....(((((..(((....)))))))).....)))))))))))))))))).

>cro-novel152

AGAUAGAAAGGAUUUUAGGCUGUUGAUUUUUACUUCUAAGGGCUGAUUCUAAAAACUAAUGCUAAAGAUU
GAUGUCUAUCC

.((((((...((((((.(((.((((.((((((..((........))...)))))).))))))).))))))....)))))).

>cro-novel153

CAUUGAUUAGUACCACGAGAUCUGUUGUUGUUUAAGGGCCUUGUCGUAUUAAUUGAUA

.(((((((((((.(((((..((.(((.....))).)).))))).))))))))))).

>cro-novel154

CGAUUCUGUAUGAAUAAGGUUCUAACUCGAGUAAGAGUCAGAUUCUACAGGAUUA

.((((((((.((((..((((((.((....)).))))))..)))))))))))).

>cro-novel155

AUCGGUAUCUUUUAGUACAUCUUUUAGCAUAAAUAAAUCCUAAAACUCUAAAAGGUAAAAAGAAGUGUAU
UAAACGAUCCUGAG

.((((.(((.(((((((((((((((.((.........................)).)))))))..)))))))).))).)))).

>cro-novel156

CGAAUUCGAGAAAUACAAGACUGACAUGUGAUAUUAAAUUGUUUCUUAAAUUACUCGAAUUCA

.(((((((((.(((..((((..((((.............))))))))..))).))))))))).

>cro-novel157

CGGAUUUCAUAUCAAAUUUGGAAUAUAUUUUUGCAUUAUAUAACCGUUUUAAUAAAUCCUUUUUAUUUG
GAAUAGGGUAUCGAAUCCA

.((((((.(((((..((((.(((((.........((((...........))))..........))))).))))..))))).)))))).

>cro-novel158

AAGGUAAUCUAUAUUUAUAUAGGUAGGUCUGUCAUCUUAAUAAAAUUAUAAGGUCUCUUUAUAAAUGUG
UAGAAACCUA

.((((..(((((((...((((((.((((((....................)))))).))))))...))))))).)))).

>cro-novel159

UGUUAUCAAAUGGCAAAAUCUCUCACUUCCAAGGUCACCACUAGACUGCCCGACGUGUAAGUAGAGAAUAU
AAAUAAUGGUGAUU

.(((((((..........(((((.((((.((..(((...............))).)).)))))))))..........))))))).

>cro-novel16

UUUCAUCAAUUCCUUCCUCUAAUCUCUGGUAUGACAUUCUUAGGUUCCCUGUCGGAUGUGUAACACUGACA
CAAUCUUAGAUGGUAUGGUGCCUUCUCUUAAAGGGUAGAAUGUCAUACCAGAGAUUAGAGGAAGGAAUUG
AUGAAU

.(((((((((((((((((((((((((((((((((((((((.((((.(((((((((((((((.......)))).))))..)))))...)).)))).((((...)))).))))))))))))))))))))))))))))))))))))
))).

>cro-novel160

UUGUGAGGAUGGGUAUAAUAAGAGGGUUUAUGUUCCUCUUCGGAUAACACCUGAGCAU

.(((.(((.((..(((...(((((((.......)))))))...))).)))))..))).

>cro-novel161

UCUUUUCUAUUGUUAAUUGUGUCUUUAAUUACGGAGAUAAGAACAACAAGAAAGGU

.(((((((.(((((..((((.(((........))).)))).))))).))))))).

>cro-novel162

ACACUCAUGACUUUGUAUGGAAGUGCAAUGCCCUAUCCCGUAAACACCUAGCAUGACUUUAAAACUAAGGA
CGUGAUGA

.((.(((((.((((((...(((((.((.(((..................))))))))))...)).)))).))))))).

>cro-novel163

AAAUGAUCAGCCUGAACGUGAAUGAUUAAAGAAAAUAAUUACUCCUUAUAGGUUGGACGUUC

.((((.((((((((...(.((.((((((.......)))))).)))...)))))))).)))).

>cro-novel164

GCUAAUCGGGGCUAAGAUCCAGUCAAUCGAAAACAAUAAAUUAGGAAAUUAAGAGUAAGAGGGAUCUUGAA CUAAGAAAUAGA

.(((.((.((..((((((((......((.......................))......))))))))..))..))..))).

>cro-novel165

AAGGUUCAAAUGUAAAGAUCUGAUUUAUGACGGUAUAAUUAAAAGUUCAGAUGUUUAGAAACUUG

.(((((....(.((((.(((((((((.(((........))).))).)))))).)))).)))))).

>cro-novel166

CAAUAAAUUAGUUUUUUUCAAAAUAGAUGGAUUGAGACGUAAAUGAUGUUAAAAAGUAUAUUUUAUCCUC UUACAAAUUAAUUUAUUA

.((((((((((((..........(((.((((.((((.(((...............))).))))))))))))...)))))))))))).

>cro-novel167

CGUAUCUUUCGAUCAAUACUGCGUUACUUAAAUAUGUUAAGAAUCUCGUAUGACUAGUAUUUGAUAAAUA AUUACU

.(((.......((((((((((.(((((((((.....)))))).........))))))))).))))).......))).

>cro-novel168

GAUUCUUUUGUAAUGAAAACUUUUGAAAGUAAUACGAUAAGAGAUUGAUUUGAAGAGAGUA

.((((((((..(((.((..((((((...((...))..)))))).)).)))..)))))))).

>cro-novel169

AGGACUGCCUAAGUCUUUAUAAAUUGUAAUUAUUAUUUUUAAUUAGCCGAUAUAAGAUUCUCUUUCUCAA CAGUUCC

.((((((....((((((.(((.....((((((.......))))))......))))))))).........)))))).

>cro-novel17

GUUACUUAAGUCGCAUUAUAACCGAAGUCAUUUUUUUUCCCUUAACUUAAAAUCAUUUGUCAAAAAGUA AUUUACCACAUGCAUCUUUUUUUGCGGGCUCCACCGGGUUAGUGGUGGCAUGCCACUUUUUGAUACGAUU UUAAUUUAAGGGUAAAAAGACAAAAGACGACUUUGGGAUAUAAUGCGAUUUAAGUAAA

.((((((((((((((((((.((((((((.((((((((((((((((.((((((((...((((((((((...........((((........))))(((.((((((......))))))...)))))))))))))).)))))))).)))))))).)))))))).....)).))))))))..))))))))))))))))))).

>cro-novel170

GUAAUAAAUAAAGAUCUACUUGCAUUACUGUACUCAUCGUAUUGAAGUAAAUUGAUUAAUUGUUAG

.((((((.(((.(((.(((((.((.(((..........))).))))))).)))..))).)))))).

>cro-novel171

AUGCAUCUGUUAUUCGUCUUUGGAACAAACCACCACUGGUAUAAAAUUUUAAAUGAGAAAAACAAGUCCAC

.((.((.((((.(((.((((((((...((((....))))......))))))).))))).)))).)).)).

>cro-novel172

UGUAUUUUUGCGUAUCCUCUUAUUUUGAAAAAAUGGAAACUUAGGUAAGUGCUUUAGAAAUAUA

((((((((((.((((...((((((((.(.....).))))..))))...))))..))))))))))

>cro-novel173

AUAAGAUCAAUUAUAAUGUAAGACUGAAUAUAUGAAACUGUUUAUAAAAUGAUUCGAACUUAA

.((((.((((((((..((((.(((...............))))))))..)))))).)).)))).

>cro-novel18

AAAAAUACACCAUGUGGACAGUUGAGCCAUGCGCUGGUGCGAGUAGGGUAAUCCCACACCAAGACUUGGUG
UGAGGAUACCCUUACUCGCGCAAAGACGUGGCUUAAGUGUAUAUAUGGUGUAUUUUC

.((((((((((((((.(((.(((((((((((....(((((((((((((.(((((((((((...)))))))).)))))))).)))))))))...).)))))))))).))).))))))))))))))).

>cro-novel19

AGUAUGACAAAAUUGUACUCAACGUGAUACUUUUUCGAUACAUUUUAGGUUUGUGUCAUGCACAUGAGUC
UUUUAUUAAAGAAAUUUUAGUUUUAAAGUGGUAAUUUAAAAAAAAAAAAUUAGAUGAUGUGUACUAGAA
GAUGAUAGAGAUAUGUAUUUGUUGAAUGCGUCCUUUUAGUUAUGUAGCUGUAUUUUAAUAAGGAAUAAU
GUGCAGCAUACCUAAAAUAUAUUAAAAAUUAUUGCGUUGAGUAAAUUUUGUCGUACC

.((((((((((((.((((((((((((((.(((((.((((.(((((((((.(((....(((((((...(((((.(((((((.((((((..(((((((.......)))))))....))))))).....(((((.(((((((((((...
..((((......)))).....))).))))))))))))).......))))))))))))...)))))))))).)))))))))).))))))))).))))))))))))))))))))))))))))).

>cro-novel2

GCUUCUGUCGUUCAUUCGUGACUACGGUUUCAGCAAGGGGGUUGUCAACAGAGGGCAAGGAUCUUCAGGC
AACACUGGACAUAUAAAUUUUGUAGAGAGAGAAAGGUUUAUGUUUAAAAUUAAUUAUUUUUGAGUUACCC
UUAUAAAUUUUCGAGUUCGGGGUCGAUAAAGAUAACUCGGAAAUCUAUAAGGAUAACUCGAAAACUUUAU
AUGUCUGGUGUUGCCUGAAGAUCCUUGCCCUCUGUUAACAACCCCCUUCUUGAAACUGCAGUCACGAACGA
ACGAUGGAAGA

.((((((((((.((((((((.((((((((.((((((((((.(((.....(((......))).....))))))......((((((((((.(((((((
.((((((((((....(((......)))))))))))))).)))))))).)))))))))))..))).))))))))))).)))))))))).)))))))))).)))))))))
))).

>cro-novel20

UGUUGAAAUUCUUACACUGUGACUGUUAAGAUUCUAAACUUGAUUCUUGAUACAAACUUCGCAGCCUCAG
AAAUAGGGUUUCUUUUGUUUGCAAAUGUGAGAAUAGAGACAAAUUAUAGAAAAAGGAGAAAAAAGAAAAG
AGGAAGGAAAGAAAAAAGAGCAUUUUACAACCCAAAUAAUAAUAAUAAUGCCUGCAUGGGUGUGGCUCAGU
UGGUUGUGGGACUGCAGAGUUGGUCCCAAAAGUCAAGGUUCGAAUUCUAACAGCGGUAGUGUGGGAAUUU
CAAUU

.((((((((((((((((((..((((((.(((((.(..(((((((.(((..((.(((((.((((.((((.((.....((((((....(((((....)))))...))))))......................................
.....((((....((((.((((......................))))))))))))......)).)))).)))).))))).))..))).)))))))..).))))).))))))..))))))))))))))))))).

>cro-novel21

GUGGUUUUUUUUUAGGUAAGGCCUAGGUUCUAUUAUUAUGUACUAAAGAGGCCCAUUUUUUUUAAAAUC
CUGGAAGAGACUAUAACUCACAACCAUUGUGCGUUAUCAUUUAGAAUAACACAUAUUGACUGUGCAUUAUG
GCUAGAAUCGGAUUAAAUAAAAGAUUUAGGCCUUUUUAGUACCCAGUUUUUAUAUUUGGGCCCAACCCAA
AAAAAACCAA

.((((((((((..(((..(((((((((......(((..((((((((((((((.(..(((((((.(((((......((.((((((..((((..((.((((.(((((........))))).)))).))..))))..)))))))).....
.)))))...))))))). .).)))))))))))))))..)))......)))))))))..))).))))))))))).

>cro-novel22

AUUUGAGUUACGCCUUGACGCGAGUAGGGAUAUCCUGCGUCAAGUUUUGGCUUAGACAAUGAGAGAAUCG
UGCUAAUGCCUGAUGCAGGGAUACCCCUACUCACGUCGGGGCGUGGCUCAAGU

((((((((((((((((((((.((((((((.((((((((((((......(((((((.((.(((.....))))))))))).)))))))))))).))))).)))))))).)))))))))))))))))))))

>cro-novel23

ACCUCCACUCUUCAGUCUCAGCCAUCCACUUCUGGAGGAUGAGUCUUUGAAGCUCCAGAAGUGGAUGGCUG
AGACCGAAGAGUGGAGGG

.((((((((((((.((((((((((((((((((((((((...............))))))))))))))))))))))))).))))))))))))).

>cro-novel24

UAAAAUUCUAUUAUGGACUCUAUACAUGUCUCUAUUCAUGGACUAUGUGAUUUGUAUUAAAUCACAUCAC
CUUUAUAGUUAUUUUUGUCUCAAUAAUAUGAGAUAUAAUUAAUAACAGAACGUGAUGUGAUAAACACAUG
CCAUAUAAUAUAUGAAUUGAGAUAUGGACUGAGUUCAUAAUAGAAUUUUC

.((((((((((((((((((....((((((((.(((((((...((((((...(((.....((((((((((.(((...((((((..(((((((......)))))))).....)))))).))).))))))))))....)))...))))))
...))))))).))))))))....))))))))))))))))))).

>cro-novel25

UUUUUAUAUGCCUAAACUAUUUUCGUGCGUCUAUAACUAUCUGAUACUAUAUUCCGUGGUGUAAUCCAGC
CAACAUUCUCACGUGACUCAUGUGGAUUUGGUUGAAUUCCGAUGCGAAAUGUGAUGUCAAGACAGUUUUA
GAUGUACGAAAAUAGUUUGAGUAUGUAAAAU

.(((((((((.(((((((((((((((((((((((.((((.(((((((.((((((.((((.((.(((.(((((((...((.((((((...)))))))).))))))).))).)).)))).)))))).)))).))).)))).))))))
))))))))))))))).)))))))))).

>cro-novel26

AGUUUGUAAAGACGUGGCUAGGACAUUAUUUGUCUAUGAAAGACUUGGGACAUAUAUUUAGGGUCUAU
GAUCGUCUGUACCACUUUGUGUAUCACUUUUUAGCCUUUUAAAGUUUAUCUUUUUCUCUUAGGGUUUAU
CUUUUACUUAUUAGGUUUUAUUAAUAAUUUAUAUUAAAUUAGAAAGUGGCACAGAAAGUGUACACAAAGG
GAUACAAACAAUCCGAGCCUAUAUUUUAGGCGUUAAGAUAUAUAGAGAUAGAUCACAUAGAUUACUUGGCC
UAAGUCAGAGUACAUACA

.((.((((..(((..(((((((.........(((((((...((((......(((((((((((.(((((.(((.((.((((((.(((((((((.((((((((.(((((((..((((((......(((..((((...........)))).
.)))..................))))))..)))).)))..))))))))))))))))))).)))).)).)))............))))).)))))))))))......)).)).)))))))..)))))))...)))....)))).)).

>cro-novel27

GUGUUUCCUGAUUAAAAAAAUAUAUAUUACCGAUCUAAUAAGUAUGAAUAAAGAAUGUAAGAUCUUUUGA
UCGUAUUAAGUGACAUUAUAUAUAUAUAUGAUUGGAAAAUUCUGUUAUAAAUUCGAUCCAUGUAAUGUG
GUGGGAUAUGUUGGGUCCAUGAGUCAAUUUUUCUUUAUGGUUAGUAUCUUUGAUUUUAAUGUAUAUUA
UGUCUCACACUUCAAAAUAUAGACGUAACUUAAUAUGAUUUAAAAGGUUUAUAUUCUUAUUCAUGUGUCA
GAUCAUAACAAAAAUUUUCUUAAAUAAUUAGUGAACAA

.(((((.(((((((..(((((..........(((((.(((..(((((((((.(((((((.((((((((((((((((((((.((....(((((.((((((((((((..............)))))).)))))))))))((((((((
((.((.......(((((((.....((.........)).....))))))).......)).))))))))))................)).))))))))))).)))))))))))))))))))))))))))))).))))).........)))))...
...))))))).))))).

>cro-novel28

GACCUAUUUGAGCAACGUCUUGGCGCAAGUAGGAGUGACCACACGCUAGGGAUUGACGCUGACCUUGAGAU
GAUCCUGUCAGUCAUUGGCGCGGAACACAUCUACUCGUGCCAAGACGUGACUCAAGUGGGUA

.((((((((((..(((((((((((.((((((.(((.((...((((((.((((((((..((.(.......).)).)))))))))).)))))).))..))).)))))).))))))))))))..))))))))))).

>cro-novel29

UGUAAUAACAAGAAAGCACAGAAUGAACAGGGAUUUGGAACUCUUCUCACUCAGUUAUGGACUGAUAAUAG
UGUUCUUCUUGCUUUUAGCUGGAGUUAUGAAUCCCUGUUUAUUCUGUGCUUUCUUGUUAUUACC

.((((((((((((((((((((((((((((((((((..((((((...(((((((((...))))))).....)))........(((...))).))))))..))))))))))))))))))))))))))))))))))).

>cro-novel3

UUCGAGUCGGUGCGAUUUUUUAAAUUUUAAACCGAUAACAUUGUAGAUAAAUGAGUAUAAAUCGAUGUAU
UAACUUUUUCUUAUUUUUAACUGUAGGAUCUUACUAGAUCUAAGAUUGAAUUAAUGGGUUUAGAGAAAA
GAUAGAGAGUGAAAAAGGAAAUUUUUGAAUUUCUUUUUUUGCACUAAUCUUUUAAAUUUCUAAGUUGAU
UUUAUUGUUAAAAAAUGUGUGAGCUAAGAUAGGGACCCAAUAAAUAUAACUCGAGAAUCUAUAAGGAUAG
UGCGAAAAAAUUAAAAAAAUUUCAUUUUCCUCCACCAACUUCCUCUCUCUUAAACAAAUCCUCUCCCCUAUC
UCUAAAAGUUUAAAUUUCAACAUCAGAUUUAAGAAGAUCUGACCGUUAAAAAUAAUUUUAAAAAAAGGUU
AUUACUUCGAUCUAUUAUUAUUUAUUUACAAUGUUAUCGGUUUAAAAUUUGAAAUAUGCAUCGGAUUGA
AA

(((((.(((((((.((.(((((((((((((((((((((((((((((((((((((((..(((.(((((.(((.(((((((((.(((((((((((.((.(((((((..(((((((.(..(((((((((....((((((((.((((.(
(((((.(((((..(((((((((.......((((((((((((.(((((.....((((.(((((..(((((((........(((.(......).)))......)))))))..)))))..)))).....)))))))))))))))))....
))))))))).))))))))...........))).))))..................))))))))...))))..)))))..).)))))))..))))))).)).)))))))))))).......))))))))).))).))))).)))..))))
))))))))))))))))))))))))))))))))).))))))))).))))).

>cro-novel30

AGUCAAAAAUAAUUUAUACGUUUUUUAAUUACUCCCGACAAGCCCAUGUGAUGACUUGUUAUGUCAUCACA
UGGGCUUGUCGGGAAUGGUUCUAAAUGGAUGAAAUUAUUUUUGACG

.(((((((((((((...(((((...(((((.(((((((((((((((((((((((.......))))))))))))))))))))))).)))))..)))))....)))))))))))))).

>cro-novel31

AGGAUCGUGUGUAACAUUUCCUGUAACAUCUCUUUGUAACAUUUCAUCUUAUAAUAUAUUUUCUCCCUUA
GGGUUUACCUUUUGCCCCUAGGGUUUAUAUUUUAUUUGAUAUGUUUUAUUAAUAAAAUAUACCAAAUUA
UGAAUGUUACAAAGAGUGACAUAGGAAAUGUUACAGAAAAUCCA

.((((..(.(((((((((((((((..((.((((((((((((((.(((.....((((((......((((..((((.........))))..))))..))))))..(((((.(((((((((....))))))))).))))).))))))))))
))))))))))..))))))))))))))))).)..)))).

>cro-novel32

AUUUGUAUACUUGAGUCACACCCUGGUGCGAGUAGGGGUAUCCCGCGCCAAGGCCUGGUGUAGGGAUACU
CGUACUCGCGUCAGAACGUGGCUCAAAUAUGCAGAG

.((((((((.(((((((((...((((((((((((.(((((((((((((((.....)))))).))))))))).))))))))))))...))))))))).)))))))).

>cro-novel33

UUGGAGGGAGUUGAAAGAGAGGGCAGACUCGCGUAAGAUAGACCGUGGGAAUCUUCGAAUCAAGCAACUU
GGUUGCGCUUGCUCUUCUGCUUCACAACUGUUAUUUGCGUGCUUCGUACGGUCUAUCCAACGCUAAUCUG
CAAUCUCACCGCUCUCUUCAU

.((((((((((.(...((((..(((((...((((..(((((((((((.(((.(..((...((((.(((..(((((.(...((......))..).)))))))).)))))).).))).)))))))))))..))))...)))))..)))).
).)))))))))).

>cro-novel34

AGAGGUUCAGUACGCAGGAGAGAUGGCGCCGUCGACUAGUUCAGUCAGUUUCUACAGAGAUUUUAGACUG
GAAAGAUAAGACUUGUCGAUGGCAUCAUCUCCUUGCGGACUGAACCUUG

.((((((((((.((((((.(((((((.(((((((((.(((((((((((((((....))))))...)))))).........)))).))))))))).))))))))))))).)))))))))).

>cro-novel35

AAAAAUUUUAUUAUGGAUAAGGUUCAUCUCCUAUCUUAUGGACCUUUUAGCUUGUAGUAAGGUACAUCAU
CCCUCCUUGGAAUUCUUGUUUCCGUAACUGAAGAUAUGAUGAACAAGAAUAUUGAUGUAACUUAAUACAA
AGCAAGAAGUCUAUGAGUAGAGAUACGGAUCUGUUCUAUAAAAGAAUUUUC

.((((((((.(((((((..(((((((((((.((.(((((((((.((((.(((((((.((((.((((((((((......)))((((((((((.((((........))))..))))))))))..))))))).)))).))).)))).)
))))))))))))))))))..))))))..))))))).)))))))).

>cro-novel36

CGAGACAUGAACCUCUUGAACUAUAUUAACCCACUUCAUUCAUGUGGUUAAUACAUUAUCUUUUUUAUAU
GCAUAAAUGAAGAUAUAAUCUACAUAAGUGACGUGGCUUAAUAUAGUUCAAGAGGUUCAUAUCUCA

.((((.(((((((((((((((((((((((.((((.(((((.(((((((((......((((((((((((....))))).)))))))))).)))))).))))).)))).))))))))))))))))))))))).)))).

>cro-novel37

UAAUGUUACAGAGAGUGUUACAGGAAAUGUUAUACAGGAUCUGAACCGGUUUGAUAGAGACUAUGAGGGC
GAGAAAUUUGACUGUUCGGAUCUUUUAUAACAUUUCCUGUCAUACUCUUUGUAAUAUUC

.((((((((((((((((.((((((((((((((.(((((((((((((.(((.((((...)))).))).))............))))))))))).))))))))))))))).)))))))))))))))).

>cro-novel38

AUGGCAUAAUCCGGAAAAAAUAAUCACGCUCAAGUUAUGUGGUCACCAUAACAAAAUCAAGCCAAAGACAAU
UGCCAUUGUUCCGGUGAGCACGUGACUUGGGCAUGGUUAUUUUAGCUGGAUUCCGCCAC

.((((..(((((((..((((((((((.((((((((((((((.(((((..(((((...(((...........)))...)))))..))))).)))))))))))))).))))))))))..)))))))..)))).

>cro-novel39

UUGAAAAAUUAUGUUAUGAAUCUGAUUCACAUCCUCAUUCAUGUAUCGUGUAGUUUAUAUUAAUUAAUAG
CACAUCACCUCCGAUUGUUGAUUAUGUCUUCAAUUAUGGAGAUAACGGCUUAAAAUAGACCAAAUGAUUUA
UGAGGGGGACAUGGAUCAAGUUCAUGACAGAAUUUUUCAC

.(((((((((.((((((((((.(((((((..(((((.((((((.(((((...((((((.(((......(((.......(((((((((..(((...)))..)))))..)))).......)))))).))))))...))))).))))))))
)))..))))))).)))))))))).))))))))).

>cro-novel4

UCUUCAUAAGUUGGACACACCUCGAAGUACAUAUCUCUACUUCUCUCUCAUUCUCUCUUGUAUUAUAAUUU
UGCUGAUCAAUAAGAAGAAGGCAAAGGAUGUUGUUAAGAUCCUGAUUUGUCCGAUCGAGAUAAGAAUCUA
UGCAUAAUUAUAAUACAAGAGAGAAUGAGAGAGAAGUAGAGAUAUGUACUUCGAGGUGUGUCCAACUUAU
GAAGA

((.(((.(((.........((.((((((((((.((...)).)))))...))).))..)).........)))...))).
))

>cro-novel40

ACUGAAAACUGCAUAUAUAGGAAUGCAUCAAUCAUGCGGUUCGGAUGCUCUGUAACAUUUUCUGUAACACU
CUUUAUAACAUUACUAAUUUGGUGUAUUUUAUUAAUAAAACCUACCAAAUAAAAGAUAAACGCUAAAGAAA
AAAGAUAAACCCUAAGGGAGAAAACAUAUUAUAAAAGGUAAUGUUACAGCAAAUGUUACAAUGGAUCUUGA
GUGUCAAUCAUGGGCCUCCUAUGAGUUAUGUUUUUUUAGC

.(((((((..((((((((((((..((.(((....((((.((.((((.(..((((((((((.((((((((((.(((((((........((((((((..(((((....)))))..))))))))............................
.(((...)))..........))))))).)))...))))))).))))))))))..).)))).)).)).))....))))).)))))))...))))).))))))).

>cro-novel41

UGUCCGCAGGAGAGAUGGCGCCGUCCGUCCGUCGAUGACUGACUGAUUCCGUCAAGAGACUCUGUUUUGAC
CUGUCCUGUCGUGGACGGCGUCAUCUCCUUGCGGACU

.((((((((.((((((((((((((........(((((.(((.(.....((((((.......)))))))).)))..))))))))))))))))))))))))))))).

>cro-novel42

UAUAUUUGGAUACAAGAUUCUCUGUACCACUUUGUGUACUGCUUAUGUACCACUUUCCUUUUAUAAUAUAUUUUCCCUUUAGGUUUUAUCUUUUUAUCUUAGAGUUUAUCUUUUAUUUAUUAGGUUUUAUUAAUAAAAUAUACCAAAUUAGAAAGUGUUAUGGAGAGUGGUACACAAAGUGGUAUAGGAGAUCCGAACCCAUAUAUU

.((((.(((......((((.((((((((((((((((((((((((.((((.(((((((..................((((((.............)))))))...........((((((((......))))))))............)))))))).)))).))))))))))))))))))))))))).))))....)))).)))).

>cro-novel43

CCAGUUAAGUAGAGGACAUAGAUGACAAUAUAGUACACAAACUGAUGUUGUUUUGAUUAGGUAGUUUUUGAAUUAUAUGCGCAUUUUCAAGUACUAUACUGUCAUCUAUGUCCUCUGCUUAACUGA

.((((((((((((((((((((((((((.((((((((.......(((((.((..(((((.((......)).)))))...)))))))).....)))))))).)))))))))))))))))))))))))).

>cro-novel44

CCAAGGCAUUGGUUGUUCUUCUCCCUCAAGGGCUUCCCUAUAUAUUAUGGGCAUACUGCUACUUUGCUUGCUUAUAAUAAGGUUAGAGAGGAGCCCUUGUCGGGGAAACAACAGCCCCAUGUCUUGC

.((((((((.(((((((.((((((..((((((((((((((..(((((((((((....((......)).)))))))))))....))).).))))))))))..))))))..)))))))..)))))))).

>cro-novel45

AAUAGAUUACCGACCGAUAUUUUGAUCGGUGAUCGGUCGAUAAUCGAUUUAGUCAGUAACUAGUUGUAACGGAAUAAAAUGUCAGUUGAUAACUGUUGCUAUCGAAUAUUUAGGGUUACCGAUCGAUUCUUUGGUCGGUAACUUUAUC

.(((((((((((((((....(((((((((((((....((((.(((((......((((((.(((((((((.((........)).))).))))))))))))))))))).))))...)))))))))))))....))))))))))).)))))).

>cro-novel46

AUGGAUUUCUUGGACCACAUUAAAUCAUAUUAUUUAUGCAUGUAGUCAGUGUUUUGGAUUAAGUGACUAAAUUAGACCUCCACUUAUGAAGAUUCAAGGUGUUAUAUUACACAACAACAUGAAUGAAUAAUGUGGUUUAAUAUGGUUCAAGAGGUUCAC

.(((((((((((((((.((((((((((((((((((((.(((((.((......((((((((((((...............)))))).....))))))))((((......)))).)).))))).))))))))))))))))))))).))))))))))))))).

>cro-novel47

UUGUACCGUUAGUUGAUUGAGAAUUGAGUACCUCAAGUUUAAUUUUAAUAAUCAAAUAUCUAAAUUAAGGUUCUGUAAAAAAAAUGAAACUUUUACUUUUGACAAUCAAGUACUCAAAUUAGGAUUUAAUCUUCUUUUUAUAGAACUUGAAGUUGAAUACUUGAUUGUUAAUAUUAACAGUUCAGGUACAAAAUUGACAAUUGAUUAAAAGUACAU

.(((((..((((((((((..((((..((((((.((.((((((.((((((((((.(((.(((....(((((((((((((.((.((((.(((((..(((((...........))))).))))).)).)).)))).))))))))))))))....))).))).))))))))))).))))).).)).))))))..))))..))))))))))..))))).

>cro-novel48

UCCAAAUCUUUUUACUCAGUAUCGUCUUGUACAUUCUGUAUUAUACUCUUUAGUACACUUCUUUUAGUAA
UAACUUUCUCUUAGAAUUUAUCUUUUAAAAUUUAAAAUUUAUUUUUUGCUUUAUAAAAUUUUUAUUAAU
AAAAUUACUAUAAAAUGUGUAUAUUAAAGAGUGUACUGAAGAUGUAUUAUGAAGAUGUAUUAGUCGAUCC
UGGCUAGAAUCUUUUGGU

.(((((...(((((.((((.((((.((.((((((.((.(((.(((((((((((((((((..(((((((...((.......(((((..((..((((((...))))))..))..))))).((((((.((((((.......))))))..
))))))..))...))))))))))))))))))))).)))).))).)))))))).)).)))).)))).))))))...))))).

>cro-novel49

CUUUUAAGGCGGUUCGGAUCCUCUGUAAUAUUUACUGUACAUUCUUUAUAAUAUUUCUAAUUUGAUAUA
UUUUAUUAAAAGACUUAUUAAAUAAAAGAUAAACCUUAAGAAAAAUUGGUAAACUCAAAGGGGAAAAAAU
GUAUUAUAAAAAGAAAUGUUACAGGAGAUGUCACAGAGGAUCCUGAGUGGUUUUAAGGU

.((((((((.((((((((((((((.(((((..((((((((((((((((((((((((..(((((.....((((((((((..(((((........)))))..)))........)))))))).))))))..)))).....))))))))
..))).)))).))))))..))))).))))))))))).)))).))))))))).

>cro-novel5

UCUUCAUAAGUUGGACACACCUCGAAGUACAUAUCUCUACUUCUCUCUCAUUCUCUCUUGUAUUAUAAUUA
UGCAUAGAUUCUUAUCUCGAUCGGACAAAUCAGGAUCUUAACAACAUCCUUUGCCUUCUUCUUAUUGAUCA
GCAAAAUUAUAAUACAAGAGAGAAUGAGAGAGAAGUAGAGAUAUGUACUUCGAGGUGUGUCCAACUUAUG
AAGA

(((.(((...((((..((....((..((.(((...(((((.........)))))))))).))....))..)))).))).
)))

>cro-novel50

UGGAUCCCAUCUGGCGCUGACCUGUAGAUAACGCACAAUGACUAUGCGUUAUUAACCUUAAUAACGCAUAU
CCAUUAUGCGUUAUUAAGCUUAUAACGUACAGUGACUGUAGCGUUAUCAUGUUAUAAAAAAACCUUACUG
GACCCACUGCCGCCCCCUUCCU

.(((.......(((((.((.((.((((((((((((((.(.(((.((((((((.((.((((((((((((((.....)))))))))))))).))))))))))).))).).))).))))))))................))).))...)).
)))))......))).

>cro-novel51

AGGUCCAUUCCAUUUUGUUAUACAGUGAAAUCGUGGUCGAGAAGUGGUAGGCUCCGGAAAAUGAUCUUCU
UCAUUUUCUCAACCGCGUUUCGCUGUAUAACGAGAUCUAGUGGAUCC

.((((((((..(((((((((((((((((((.((((((.(((((((((.(((.((........)).)))..))).)))))).))))))))))))))))))))))))))..))))))))).

>cro-novel52

UUCCGGCAUCACAUUUUGCGGUAGAAUUUGGCCAAAACCACAUGAGCCAUGUGAGGGUUUUAGCCGGAUU
CUACUGUGAAAUUUGGUGUCGGGU

.((((((((((.((((((((((((((((((((.(((((((((((...)))))...)))))).)))))))))))))))))))).)))))))))).

>cro-novel53

GUGCUGGACAUCUCAAAGAUCUGGCACCAGUUCAUUGUUUUUCUUCAGUUUCUGGGGAAAUUGGAAACUU
CUGGUGCUGGUUCUUGGAGAUGUCUAGCGU

.(((((((((((((.((((.((((((((((.........(((((((((...)))))))))..........)))))))))).)))).))))))))))))).

>cro-novel54

ACUCUUAGGCUCAACUUGGAUUGGCUUUUAUUUUGAAAACUCUUCCCUUUUUCACUUUGGGAUUCACAU
CUCCUCUCACAAAUAAUUCACCUAUCACAAACUCCAAGAUAAAAAUACCUCUUUUCAAAAUAAAAACCCAUCC
AAGUUGAGCCUAGGGAGA

.(((((((((((((((((((.((.((((((((((((((....((((............))))..............................((((...........))))..........)))))))))))))).)).))))))))))))
))).)))).

>cro-novel55

AGAGAGGUGAGACUUUGGUUUUUGCAUAAGCCAUCCAACUGCUAUACCAUUCAUUUGGAUAUGAAGAAGC
UAUAGUUGGAUGGUUUUUGCAAAAUCCCAAGUCUUUUACUUCUUC

.((((((((((((((.((.(((((((.((((((((((((((........(((((......))))).......)))))))))))))).))))))).)).)))))))..))))))).

>cro-novel56

AUGGAUUAUGGAGAUGCAGAGUUGGUACCAACGUUCAAGGUUCAAAUACUGGUAGCAGCAGCGUAGGAAU
UUCAACUUCUCCAAUGUGAGGGGUGGUUCCUUGUACGCACCAGUGUCUGGCCUCCCGUCUGCAGAAUUGCU
GAAUCACCAUGAUUUAC

.((((((((((.(((.(((((((.(((...(((....((((.((.((((((((.((....(((.(((((((...(((((((......))))))))))))))))..)))))))))).))))))..))).)))..)))).))).)))
.)))))))))).

>cro-novel57

UGGUAAUAACGCACAAUGACCGUGGGAUAUCAAGCUUAAUAAUGCACAUCCAUUAUGCGUUAUUAAGCUUG
AUAACUCAUAGUGACUGUGUGUUAUCACCU

.(((.((((((((((.(.((.(((((.(((((((((((((((((((.........))))))))))))))))))).))))).)).).)))))))))).))).

>cro-novel58

ACUGCAAUCUGAUCGUGUUCAUGGCAUCUCAUCAAUCCUCUCCCUCUCCUUUCUCCUUCUUCAAGUCUUAA
AUUGAGUUUGGUUGAUGAUAUGCCAUGAACAGCGAUCAAUUUGCAGC

.((((((..(((((((((((((((((((.((((((((((((................................)))...))))))))).))))))))))))).)))))))..)))))).

>cro-novel59

GGCGAAUUCAGAAAAGUAUACGGUGUCUUUGUGUGAAAAUCACGACGUGAGUUACACAUUACCAAUACUG
UAUAAGGAUAAUAUUACUAAUUGGCAAAUGAACACAGUGUUCUGGAUAUGUUUUCUUUUUAUGACCGAA
CCAUAAGUAAUUCACGCAAUGAUCUUUGCUUAGAAACACUGUAAGUCGCUGUAUUUGUA

.((((((.(((.......((((((((.((((.(((((.((((...((((((((((((((..((((((((((..........((........))..........)))))))..)))..))))........(((((.......))))))))))
)))))...)))).))))).)))).))))))))).....))).)))))).

>cro-novel6

ACUCUGUAUUUUUAGUGAAUUGUUAAUUGAGUAGCUACAUUUUUAAUUUGACAAAUAAGUAUCUAAAAG
GGUUUUUGUAAAAAACAAAAAUUAAUAGUUGAAAAAAUUUAACAGUUAAUUUUGACAAUCAAGUAUUCAA
AUUAGGGUUCUGUAAAAAAAAUAACAGGUUGAACCCUGAUAUGGGUACUUAUUGUUAAAUUAAAGUUUU
UCAACAAUUAUUUUUCUUUUUAUAAAAUCCUAAAUUUGAUACUUAUUUGUCAAAUUAAAAGUUUAGCUAC
UGAAUUGACAAUUCAUCAAAAGUAUAGGGG

.(((((((((((.((((((((((((((.((((((((.(((((((((((((((((((((((((.....(((.(((((((((((...((((.((((.(((((((((.(((((........(((((((((.(((((((((.(((((
(((((...................))))))))))).))))))))))))))))))))))))).))))))))).)))).)))).))))))))))).)))......))))))))))))))))))))))))).)))))))).))))))))))
)))).))))))))))))).

>cro-novel60

GGUUUAUGUUUUUAUGAAUCCCUUGAUUUGUAUUAAGCCACUCCACCUCUCUUAUUAACUAUUUACCUAA
UUAUGGAAACAAUAAUUUCAAGAAGGGGUGAUGUGACUUAAUACAAGCUAAAGGGUAUGAAUAAGGAUAU
GGAC

.(((((((((((((..(((((((..(((((((((((.(((..((((((((((((((....(((.((.......)))))...))))...))).)))))))..))).)))))))))))...))))).))..))))))))))))))

>cro-novel61

AUAUACAUAAUUUGUUCAUAAAAGAGUAAAAUAGACGUAAUAUUACUCUUAUAAAGUAAUUGUAUAAGUG
AAUGAUGUAUGUUUCUAUGCAUAACAAGACAGUUGAUUAGCAAAUAAGGGUAGUGCUCUGCCUAUUUUAC
CCUUUUAUGACCAAACCAUGUGUAA

.(((((((..((((.(((((((((.(((((((((.((...((((((((((((....(((((((.....((....(((((((....)))))))..))..)))))))........))))))))))))...)).))))))))).))))))
))).)))))..))))))).

>cro-novel62

AUGGAUAUUAGGGGUAAUGGACUUUUUUGCCACCUCUAAAUAUAAGCAAGCUUAAAAUUGACUUAUUAAU
GAUUAGAGGUGGCAAGAAAUUUCAUUAGUCCAAUACCCAC

.(((.((((.(((.(((((((.((((((((((((((((..((((((((........))).)))))......)))))))))))))))).))))))).))))))).))).

>cro-novel63

UUAUAUAUAUAUACACCUAGAAUCGUCUGUAACAUUCUCUGUAGUGUUAUAGAGAAUGUUACAGACGAUU
UAAACUGAUAUAUAUAUAUAU

.(((((((((((((....(((((((((((((((((((((((((....)))))))))))))))))))))))))....)).))))))))))).

>cro-novel64

AUAACGCACAAUGACUGUUUGUUAUCGCCAGAUCAGCGCCAGAUAAGAGCCACUCAGUUUUUACGCAAUU
AGCGCGGCUGAACGCGGCUUAAGCCGCAUGAUUAGGGAUAACGCACAAUGACUGUGCGUUAU

((((((((((.(.(.(((.(((((((.((.((((((((...(....(((((.(((((((.....(((.....))).)))))))..).)))))...)))).))))).))))))))).))).).).))))))))))

>cro-novel65

AGGAGCACUAUUACUGAGCUCACAACAUCAGGUAGAAAAUUUGAAAAGUAGAAGUCAGAUGAAGGGGGUU
GGAACUGUGAAGCCUGAUGAUGUGGGUCCGGGAUUAGUGCUCCA

.(((((((((...(((.((((((((.((((((((.....((((((..........))))))..(.((........)).)...)))))))).))))))).)))...))))))))).

>cro-novel66

UCGGAGUCCCUUUGGACGCCCACCGCUCGGCACCAUCUGUAAUAUGAAUGACGGUGCCGCGCUGUGUGCGU
CUGAAAGACUCUGC

.(((((((..(((((((((.(((.((.(((((((.((............)).))))))).)).))).))))))))).))))))).

>cro-novel67

ACUUUGUAUCGAUCUCUAGAAUUCACGUUAUUUUAUAUUUCACACUUUGUAUCGACACUUAAUGUUUUA
UUUUUAUCCUUGUAUUUAGGUAUAGUUCCGAGUUAUUUACUCCUAAAUUUCAUCAUAAUGGUGAAUUGU
UUAAUUAUAAUGAAGGAGGGUGUGUUAGAAGAACCGUACAUUACAUUGACUUGCGUACUGUUGGUAAUU
UUUGUGAGAAGAAUUUAAAAAUGACUGUUGAUUUUGAAACUGUAGAAUUAAUAUUAAGG

.(((.((((.(((.(((((((((.((((((((((...((((((((.....((((((((....((((..........((..((((.....((((.((((....(((..(((.(((...((((((.(((((.............))))).))
))))..))).))).)))..)))).))))..))))..))...))))..))))))))).....))))))))).......)))))))).)).)))))........))))))).)))).))).

>cro-novel68

AUGUCAUCUUUAGUAAAUGUAAAAUUGUUCAGAUAGAAAUAUAAGUCCCAACCUGCUACUAGAAUGGGUC
AAAAUAAACCUGACAAUGGCUUGAAUCGGUACAUGGGUCAGGUGAUUUCGAACUAUUCAAGUUUUAAUU
GGUGCCUACAUUAGUGGUUGGAUAACUUUAUACGUGAUGUUAUGUGACAC

.((((((.....((...((((((.((((((((.((..(((.((.((.((((......(((.((((((.((.((((..(((((((.((((((......))).)))..))))))).)))).)).)))))).)))......)))).)).))
.)))..)).)))))))).))))))....)).....)))))).

>cro-novel69

CUCCUCUUCUUCCUUCAUUCCAAAACUAGGCUAUAUUUGAUUUGAUUUUCUUCUAAGGGCUACAAACAAUA
AAUCAAAUGCAGCCUGAGUUUUGGAGUGAAAAAAGGAAAGGAU

.((((.(((((..((((((((((((((((((.(((((((((((........................))))))))))).))))).)))))))))))))..))))).)))).

>cro-novel7

CUUCUUUUGUAAUAUGUUUUUCUUUUUAAGGUUUACUUUUUUUCAGUUCGAAUCCUCUGUAAUAUUUCU
UGUAACAUUCUUGAUUUGGUAUAUUUUAUUAAUAAAACUUACCAAAUAAAAGAUAAACUUAAGAGGAAAA
UGUAAACUUUGAAGGGAAAAACAUAUUAUAAAAGGAAAUAUUACAAAAACAAACGAUCUUGAGUGCCUUU

UUCUCUUAAGAUUUAUCUUUUACUUGGUAAGUUUUAUUAAUAAGUAUACCAAAUUAAGAAUGUUACCGGA
AAUGUUACAAGACAAUGAAAUAGUGAAAUGUACUUAAAUUACAUUGCAUAUUGUAAGGGAAA

.((((((((((((((.......((((((...(((.((((.....((((....((.(((((((((((.(((((((((((((((((((((((((.((((((((((((((((((.((((((((((((((((((((((((.((..(
(((.((..(............(((........)))............)..))..)))))).))))))))))))).))))))))))).))).)))))))))))))...
.))))).....)))).)))))))))).......)))))))))))))))).

>cro-novel70

CGAUUCAUAUUCCCACUUGUAGACCAUGUAGUUUGUAUAAGGCCACAUCAUCCUUCUCUCUUGUUAAUCUU
AUCUCUAAAAUUGGCGUCACAAUCAAUAGCAGAAGGUGAUAUGGCUUAACAUUGGUCCAUGAGUGAAAACG
UGAAUCA

.(((((((.((..((((((.((((((((..........((((((.((((.((((((((.(((.................((((......))))))).)).)))))))))).)))))).))).))))).))))))..)).)))))))
.

>cro-novel72

CGAUCAUGGUGCACAGGAGGACGUCGUAAUUGCUAUCUCGGAAGCACAUCUCCGGCGUCCUCUUUGCACCA
UGAUUA

.(((((((((((.(((((((((((((....((((........))))......)))))))))))))))))))))))))).

>cro-novel73

UAAUGGAAUUGAGGGAGGAGACCUGUGAUGUAUGAUGAAUUGUCCGUACCAUAUGAGGAGUAUUCCUUU
GAUUUUUCAAUACCUUAUUAUCUUCUGAGGUCUCCUUCCUUUUUCCAUUU

.(((((((..((((((((((((((..((.(.(((((((..(((...........(((((....)))))..........)))..))))))))).))..)))))))))))))).))))))).

>cro-novel74

AAACUAUAUAUUAUCUUCGGUCUGAAAUAUAAGUUUUUUUAAUCAAAAUUAUAAGUCUUUUUAUACAUUU
CAAUGUGUAUUUAUUAUCUAUUUUCCAACUCUAUCCUCUUUUAAUUCAUCUAUUUACCUCUCUUCCUUUC
UUUCUCUUUCCUUAUUUAUUGAGGUACCUUAAAAAUUAUAUUAUUUCUUCAUCCUUAAUUCGUGUGUAAA
AAGACUUAUAUUCUUGAUCGAAAGUAGUAUAAGUUA

.((((.((((((((.(((((((...(((((((((((((((..((......((((.......((((((......))))))..((((((.........................
....))))))..........................))))......))..)))))))))))))))...))))))).)))))))))))).

>cro-novel75

UCAAUAUAGGAUUCUAACCAACUACUAAUAAUUAUAAUCAAUAUUAUUAUAGUUCUAUUUCUUAUUAUUA
GUAGUUGGUUAGAAUCUUAUAUUGU

.(((((((((((((((((((((((((((((((((..........((((...))))..........))))))))))))))))))))))))))))))))).

>cro-novel76

AAAAAAUAUCAUUGAGUGCAGCGUUGAUGAAUUCCAAGUUCCAAGUUUUGGUAUUUGUAUUCUCACUGUG
GAUUCAUCAUCGCUGCACCCAAUCAUAUUUUUA

.((((((((.((((.((((((((.((((((((((((((.((((...)))).)))))...........))))))))).)))))))).)))).)))))))).

>cro-novel77

UGAUUAGGUAUAACGCACAGUCAUUGUGCGUUAUCACCUAAGAUAACGCAGAAUGACUGAACGUUAUGGCC
UGUUCC

.((.((((((((((..((((((((.(((((((((......))))))))).))))))))..)))))).))))).)).

>cro-novel78

AUUAAAAUUAUUGACUGACGGUAUUCGAUCGGUAAUUAUACAUCAAUUACCAACCGAAAUCGGUAGAUAAC
UUUUGAAUAUUACCGACCGAUUACUGUCAAUUGAUAAUUUUGGC

.((((((((((((.((((((((.(((.((((((((.((.((....(((.(.((((....)))).).)))....)).))))))))).))).)))))))).))))))))))))).

>cro-novel79

UUACCCUACCUUUGUGUUACUAUCUUUUUUUAUUUAUUUUGUAUGGAAAGAAAAAUGAUAGUAAAACAAA
GGUAGGGUGC

.(((((((((((((.(((((((((((((((.(((((.....))))).))))))).)))))))).))))))))))))).

>cro-novel8

UUUUCAAAAAAAAGUACUAAAUAGGUUUUUUUUUUUUUUUAAAUCCUGAAGAGGUCAUAACGCACAGUC
AUUGUGCGUUAUCAAAUGAGAUAAUGCACAAUGGUUGUGUUAUAACUAAUCAUGGGCUUAAUCCGCGUUA
AGUGCUGAGGUGGAUAAUAACGCACAACCAUUGUGCAUUAUCUUGAUGAUAACGCACAGUGACUGUGCGU
UAUCUUGUUUAUGUUGAUUAGGAAAAAAUAAUUUUUGUAAGUAUUUGGAAAACUUGAAAC

.((((((....(((((((....(((((.(((((((((..((((....((((.(((..((((((((((((((((((((((((((..((((((((((((((((((((((((((..(((.(((.(.(((((((....))))))).)
)))..)))...)))..)))))))))))))))))))))))))).)))))))))))))))))))))))))))))).)))))..)))))..))))))))).)))))).....)))))))).......)))))).

>cro-novel80

UGAAUCGUCUGUACCACUUUGUGUAUUAUUAUGUGUACCACUUACUAAUUUCAGUAUAUAUUAUUAAUAA
AACUUACUAAAUAAAAAAUAAAUCCUAAGGAAAAAUAAUAAACCACAAGGAAAAAGUAUAUUAUAAAUGGA
AAUGAUACAAAAGGUGUUACACACAAGUGAUACAGACAAUUCU

.((((.(((((((.(((((((((((.((((...((((.((.((.(((.((..(((((((.((((((......((((..................))))......)))))).((....)).....)))))))..)).))))).)).))))
...)))).)))))).))))).))))))).)))).

>cro-novel81

AGUCCGGUCCUCGUGCGUACUAGGAUCUGAUCUCCAGUAUAUAGAAUCGUUAAGUCAUAACAAAUUAUAAG
UCCGGUCCUCGUGCGUACUAGGAUCUGACC

.(((.((((((.((((((((.((((((.((.((......(((((....((((.....))))...))))))))).)))))).)))))))).)))))).))).

>cro-novel82

CUCAUCAUAAUUAUCUUCUUUAGGCUAUACUUGAGUAAGAAUCAGAUGUGGACUAUCAGAUGACGCUAAA
UAAUAUGCAUUCUUUUGAAAAUCACCUAUCUGACCAUUAUCAUUUGUGCCACUAAUUGUGAAGGAGAAAAC
AAUGAUGGA

.(((((((..((.((((((((((((.(((..(((.(((...((((((((((....(((((.((.((..........))..)).)))))...))))..))))))...))))))..))))))........))))))))).))..))))))
).

>cro-novel83

CUCACGGUGAAAUCAAGUGUACCUAGACACAUUUGGUGCAUGUGUCAAAUUAACUAAUAUGUACUAGACAU
GUAUAGACACAUCUGAUUCUAUCGUGAA

.((((((((.(((((.((((..(((.(((..((((((((((((............))))))))))))..))).)))..)))).))))).)))))))).

>cro-novel84

CUCUAAUGUAUCUUCUGGUACACUUUCUUUGGAACACUUCUUUUAUAGUAUUUUUAUAAAUGAACUUUAU
AAAAUAGAAGAUAAAACCUAAGUAAAAAAAGAAGUAUAGAUGGAGAAAUGUAGCAAGAAGUGUAUUAUAA
GAUGUAUUAGAU

.((((((((((((.(((((((((((..(((..(((.((((((....((((((((........((((.....................))))......))))))))....)))))).)))..))))))))))))))).))))))))))
)).

>cro-novel85

AAGUUUAGAUUAUAAAUUAGGAAAUGUCUAAGGUCCAGACAAGUAGAAUGUCUAAUAGUAUAAAACGUUU
AUCAUAAUUAUCAGAAUAAAUCAAUUUAAAUUUGAUGUGUUCAGUGGGAGAAGUAUAUGUUUUACAGUU
AUCAUUAUUUUCUGUCUAAACUUGCGUCUUAAUUAUAAUUAGACUA

.(((((.((((((((.((((((..(((...((((..(((((.(.((((((......(.(.((((((((.(((.....((((..(((((.(((((.......))))).))))).)))).....))).)))))))).).)......)))))
)))))))..)))))))))))))))))))))))))).

>cro-novel86

UUAGUACAUUUUUUAAUACAUUUUCUAAUAUAAUUUUUUUAAUAGUAACUUUCUUUUAGAAUUUAAGAU
UUAUUUUUAAAAUUUAGGAUUUAUAUUAUAAAAUUAUUUUAAAAAGUAUUUAUUAGAAAGUGUACUAAA
AGAUGUCUGU

.(((.((((((((((.((((((((((((((....((((((((.(((((.(((.(((((((.(((((((.....))))))).)))))))..........))).)))))))))))))....)))))))))))))).)))))))))))
)).

>cro-novel87

CUCACAGAAUGUAUUCAGAUCGUCUAUUACAUCCUCUUGUACGUUUUUUUUAAUAUUAACUUUUCUCUUC
GAGUUUAUCUUUUAUUCUAUAAAUUUUAUAUUAAUAGAAAUACUAUAAAAUGUGUAUACCAAUAGAAGAU
GUAUUAGACGAUGGCUAACGGAAUGAAAUGGGGUGAA

.((((...((.(((((..((((((((.((((((.(((((((((..((((.((.(((.(((((........)))))........((((((.(((.....))).))))))))).)).))))..))))))).....))).)))))).))))
))))........)))))..))...)))).

>cro-novel88

AACACUCGAAAUCGUCUAAUAUACACUUUUUAUAGUUAUUUUAUUAAUAUAAUUAAAAGUGUACUAGAGU
AUAUAUUAGACGACCUUGAGUGUA

.((((((((..((((((((((((.(((((.((((.((...((((....))))....)).))))..))))).))))))))))))..)))))))).

>cro-novel89

ACAACUGUCGUCACUAUACUAAAAUGUAUACUUAAAGGUAAUGAUGAAGCAUAUAAGAAUGAGCCUAAAUA
UCUAUUGUUAUCUUGGGAUAUUCAUUUCUGUAUAACGAUGAUAGUUGC

.(((((((((((..(((((..(((((.((((((((.((((((((((..((.(((....))).))..........))))))))))))))).))).)))))..)))))..))))))))))).

>cro-novel9

GUAGCUGAAAAUAUCAAUUUAUCACCUAGAAGGCGGAAUCCGGCUGCUUCUUUCAUGUGAAACGCACGUGU
ACGCCAUGUGCAACCGUUCUUAUUGACAUAGGUACCACUCACACCAGCUGCACGUGCGUUUCACAUGAAAGA
AACAGCUGAAUUUCGUCUUUUAGCUGAUAAAUUGAUAUUAUCAGCUAG

.(((((((.(((((((((((((((.((((((((((((((.((((((.(((((((((((((((((((((((((.((..((((.....((.(((((....))))).))....))))...)))))))))))))))))))))))))
.)))))).))))))))))))))).))))))))))))))).))))))).

>cro-novel90

AUGAGGAAAGAGAGAGAUAGUGGGAGUGAAAUAAAUAUAUAGAAAGGCGGGUAAAGAUGAAAGAUUCAAU
AAAUACUUAUUGAAAAUUGUAAAAAUUUUUAUAUUUUUAACCAUUCUCAAUGAGUCUAAAACCUUUAUAU
UUUCAACCGGACGUACUAUAUUCUUGAUCAUCCUUAA

.((((((....((((((((((((.(..((....(((((((....((((..........((((...))))......(((((((((.(((.((((((((......)))))).)).))).)))))))))......)))))))))))).....)).
.).))))))).)))))).....)))))).

>cro-novel91

ACUUGCAUCAGUGUCUGGCCUUCAGUCUGUAGAGUCGCUGAGUCACCAUGAUUUACAUCUUGCACCUGAGC
GUUGGACAGUCUGGUGGGGGC

.(((.((((((((((((((.(((((..((((.((..(.((((((((...)))))))))..)))))).))))).)))))))))..)))))).))).

>cro-novel92

UAGACAUCUGCGUGGAUGGGUCAACACGGGCAGCCCAACAACUUGCUUUGAUACCAUAUUAAAAUUUGAGA
UUAGGCUUAAUUCAUUUCAAAAAAAUACUUUAAGAGGGAGGAAGGACUGCCCAACUUGAAUUAGCUGAUG
UGAGAUUCUU

.(((.((((((((((.(((.((((...((((((.((.....(((.((((.......((((....((((((((.((......)).))))))))..)))).....)))).)))...)).))))))...)))).))).)).)))).)))))))
).

>cro-novel93

ACGGUUAUGCCAGAUUUUUUUUAAAAAAAUAUUGUGAAUUUUCAUAUCAACCAUUUUCUUUGCAAGAUCC
CCAAUAUCAUGAAGGCCGAAAAAAGCUGCAUCAUCACUUGAUUUCACGUAAGAAAUGAACAUAUCACGUUG
UGCUUUUAAAGAAAAAACAUCUGAAUAUAACGCGG

.((((((((.((((((((((((((((..(((.(((((((.(((((..........(((((((..(((((.........(((..(((........))).)))........)))))..)).)))))))))).)).))))).)))..)))))
))))))....)))))..)))))).)).

>cro-novel94

AUGAUAUAUAAAGUAAUAUACAUGAUUCGAACCGAACUAGUAAAUAUGCAUAAACGAGUUUCAAUGUGCA
UGGUAAGGUGAUAGCUCACUACUAAAAACGUAUGUUAUUAAGUUACAAGUUCUGGAUUCGAAUCACAUCU
CAUCCUUCAAUAUUAA

.((((((...(((.........((((((((((((((((......((((((((............))))))))((((..(((((((..((..........))..)))))))..)))).))))).)).))))))))))..........)))..)
))))).

>cro-novel95

AUUAUAAUACUCAAGAUCGUCUAAUACAUCUGUAAUACACUCUCUACUAUACUUCCUUUUAAUUUAGAUUU
UAUUUUUUAUUCUAUAAGAUUUUUAUUAAUAAAAAUUAUUAUAAAUAGUAUGUAUUAAAAAAAAAUAUA
AUAGAAGAUGUACUAGACGAUCCGGCCAAAAUUAUAAA

.(((((((...(..(((((((((.(((((((.(((((((..((.((..(((.......(((((..(((((((((..........)))))))))..))))).......)))..))...))..)))))))................))))))
).)))))))))..).....))))))).

>cro-novel96

ACAUCUUUCACCUGAGCGUUGGACAGUCUGGUGGGAAUUUCGCUUCUUAAUAUAAAAAAUAAUCUGAUCU
GUGGAUCAAUGUUUAAAUUCUGACUGGAAAUUUCAACCUUUCAAUAUGAUAGGUGG

.(((((.(((..((((.((((((...((((((.(((((((................(((((...(((((....))))).))))))))))))).))))))...))))))..))))...))).))))).

>cro-novel97

UCGUUUGCCAGUGAGUCGACUCGAGCUCGGUCAAUAGUAAACUCAAACCAAGUUUUGACCGAGCAACCUGU
GAAUUGCUCACGAGCAACUCGC

.((.((((..(((((.(((.(((.((((((((((.....((((.......))))))))))))))))......))).))))))))..))))..)).

>cro-novel98

UUUAAUAGAAGGUAAUUGAGUUUGGUCUGUGAGUAUAAGAUUUAGAUUUUACCCGCUAUAAGUGAAUAA
GAUUUGGAUAAUGUUUAGAUAAGACUUGACUGGUUUACUAUCCCUAUUAAU

.(((((((..(((((.(.((((.(((((......(((..((((((((((((..((((...))))..))))))))))))..))).......))))).)))).).)))))....))))))).

>cro-novel99

AUAUAAUUACAAAAAUUAUUUAAUUACACUCUCUAGUAUACACUAUUUAUAGUAAUUUUAUUAAUAAAAU
CGUAUAGAAUAAAAGAUAAAUCCUAAACUUUAAAAAAUAAAACCCUAAGAAAAAAUAAUUAAUCAAAUAAA
UGUACUAAAUAGUGUACUAGAGAAUGUAUUAGACGAUCUUGAGUGCAUAUAU

(((((..(((.((.(((.((((((.(((.((((((((..((((((((..(((((..((((((...))))))..))))))))))))
))))))))).))))))))).))).))..)))..)))))

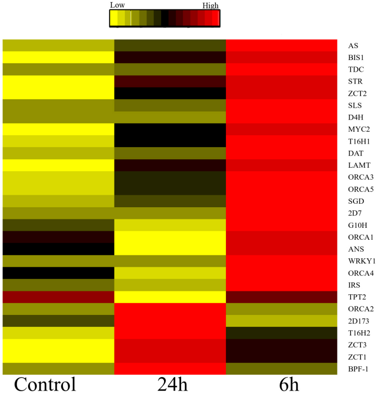

Figure S4. Heatmap of terpenoid indole alkaloid (TIA) biosynthesis pathway genes in response to different Jasmonic acid treatment regimes. The color scale represents the values of reads per kilobase of transcript per million fragments mapped (RPKM).

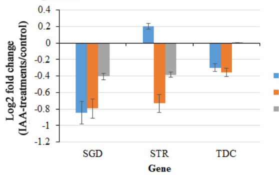

Figure S5. Quantitative real-time RT-PCR analysis of selected terpenoid indole alkaloid (TIA)biosynthetic pathway genes. The relative abundance of individual gene is presented as the ratio of auxin-treated and control samples. The mean expression value of target gene was normalized against the expression of endogenous control RPS9. The normalized expression (2-ΔCT) in IAA-treated sample was divided by the normalized expression in control sample and log2 transformed. The data represent the mean values ±SD of three replicates.

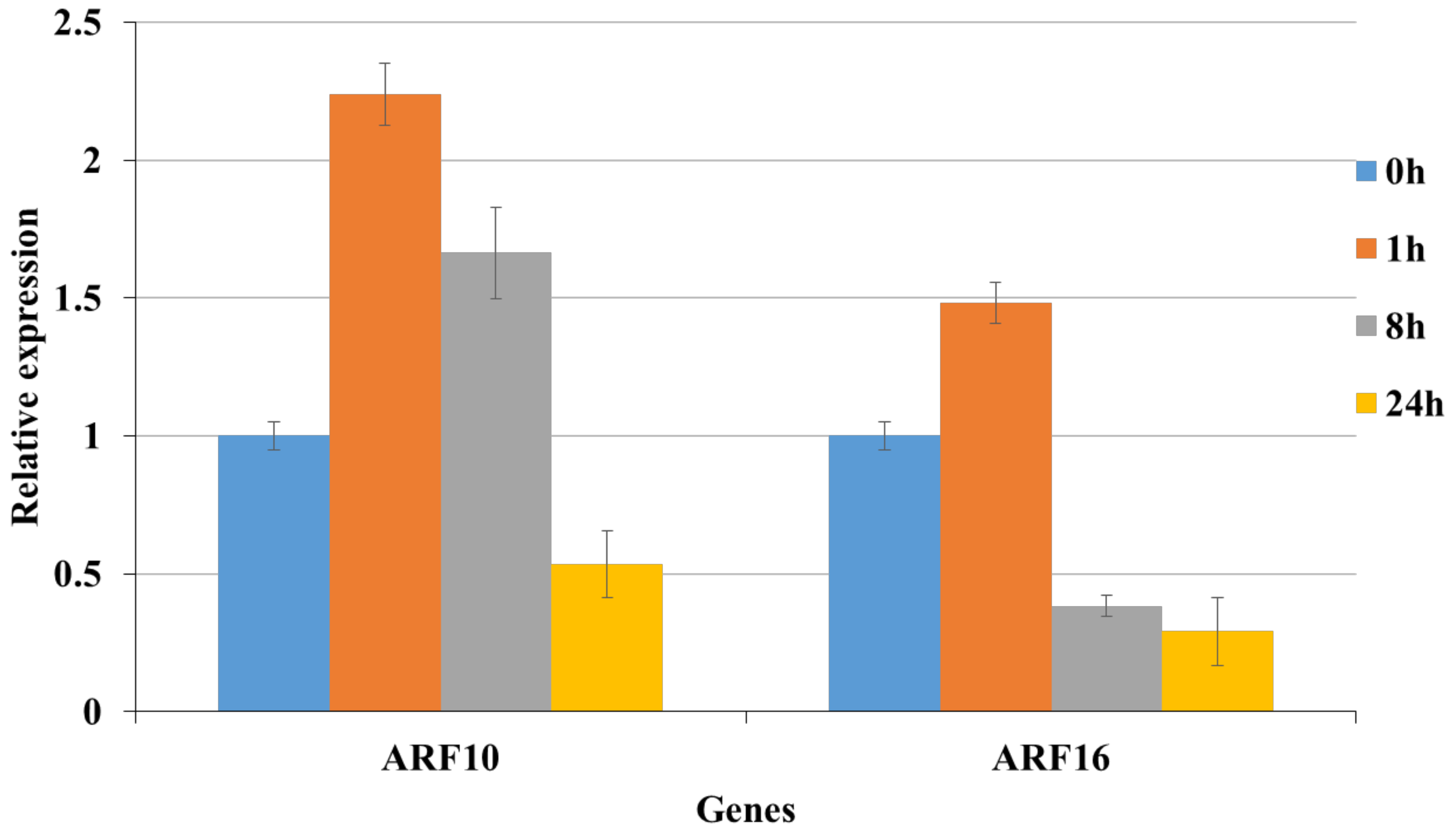

Figure S6. Relative expression of *ARF10* and *ARF16* in the control and IAA-treated *C. roseus* seedlings as measured by quantitative RT-PCR. The expression values of target genes were normalized against that of the endogenous control, RPS9. The data represent the mean ± SD of two biological replicates.
